# A comparison of methods for detecting DNA methylation from long-read sequencing of human genomes

**DOI:** 10.1186/s13059-024-03207-9

**Published:** 2024-03-11

**Authors:** Brynja D. Sigurpalsdottir, Olafur A. Stefansson, Guillaume Holley, Doruk Beyter, Florian Zink, Marteinn Þ. Hardarson, Sverrir Þ. Sverrisson, Nina Kristinsdottir, Droplaug N. Magnusdottir, Olafur Þ. Magnusson, Daniel F. Gudbjartsson, Bjarni V. Halldorsson, Kari Stefansson

**Affiliations:** 1 deCODE Genetics/Amgen Inc., Sturlugata 8, Reykjavík, Iceland; 2 School of Technology, Reykjavík University, Reykjavík, Iceland; 3 School of Engineering and Natural Sciences, University of Iceland, Reykjavík, Iceland; 4 Faculty of Medicine, School of Health Science, University of Iceland, Reykjavík, Iceland

## Abstract

**Background:**

Long-read sequencing can enable the detection of base modifications, such as CpG methylation, in single molecules of DNA. The most commonly used methods for long-read sequencing are nanopore developed by Oxford Nanopore Technologies (ONT) and single molecule real-time (SMRT) sequencing developed by Pacific Bioscience (PacBio). In this study, we systematically compare the performance of CpG methylation detection from long-read sequencing.

**Results:**

We demonstrate that CpG methylation detection from 7179 nanopore-sequenced DNA samples is highly accurate and consistent with 132 oxidative bisulfite-sequenced (oxBS) samples, isolated from the same blood draws. We introduce quality filters for CpGs that further enhance the accuracy of CpG methylation detection from nanopore-sequenced DNA, while removing at most 30% of CpGs. We evaluate the per-site performance of CpG methylation detection across different genomic features and CpG methylation rates and demonstrate how the latest R10.4 flowcell chemistry and base-calling algorithms improve methylation detection from nanopore sequencing. Additionally, we show how the methylation detection of 50 SMRT-sequenced genomes compares to nanopore sequencing and oxBS.

**Conclusions:**

This study provides the first systematic comparison of CpG methylation detection tools for long-read sequencing methods. We compare two commonly used computational methods for the detection of CpG methylation in a large number of nanopore genomes, including samples sequenced using the latest R10.4 nanopore flowcell chemistry and 50 SMRT sequenced samples. We provide insights into the strengths and limitations of each sequencing method as well as recommendations for standardization and evaluation of tools designed for genome-scale modified base detection using long-read sequencing.

**Supplementary Information:**

The online version contains supplementary material available at 10.1186/s13059-024-03207-9.

## Background

The predominant modification of DNA in humans is the methylation of a cytosine preceding a guanine (CpG), commonly referred to as either CpG methylation or 5-mCpG [1]. Accurate detection of 5-mCpG patterns is necessary to understand the complex regulatory mechanisms underlying gene expression [2], cellular differentiation [3], and imprinting [4]. Currently, the most common 5-mCpG detection methods [5, 6] do not directly detect base modifications in DNA as they rely on bisulfite conversion of DNA samples followed by either targeted methylation assays or whole genome bisulfite sequencing (WGBS). Array-based methods enable measurement of up to ~ 900,000 CpG sites [7], while WGBS has the potential to measure most of the ~ 30 million CpG sites in the human genome [8].

Bisulfite treatment of DNA converts unmethylated cytosines to uracils, while methylated CpGs remain as cytosines and PCR amplification of the DNA, then converts uracils to thymine [9]. Methods based on bisulfite treatment of DNA therefore require CpG methylation to be inferred indirectly from the sequenced DNA. Additionally, 5-hydroxymethylcytosine (5hmC), another modification found in DNA [10], is also read as methylated cytosines by sequencing methods that rely on bisulfite treatment and thus cannot be distinguished from 5-mCpG. By adding an oxidation step before the bisulfite conversion, 5hmC is converted to 5-formylcytosine (5fC), which then is converted to uracil after bisulfite treatment and can therefore be distinguished from 5-mCpG [11, 12]. This method is known as oxidative bisulfite sequencing (oxBS) [6]. These treatments negatively influence the quality of DNA samples as they can cause severe DNA degradation, thereby complicating the sequencing process [8].

With the advancement of long-read sequencing, methylation detection can be accomplished directly from the raw sequence data, offering the possibility to perform detection of a wide range of modifications without the need for chemical treatments of the DNA [13]. Long-read sequencing technologies have the capability to produce substantially longer reads, at the cost of having a higher error rate than previous short-read technologies.

Nanopore sequencing uses a protein nanopore embedded in a synthetic membrane [14]. An electrical current is applied across the membrane, leading the negatively charged single-stranded DNA to move through the nanopore. Changes in the electrical current are measured as each DNA molecule disrupts the ion flow in the pore. Importantly, nanopore sequencing has the ability to detect modified bases by distinguishing their electrical current shifts, from those of unmodified bases, measured as they pass through the pore [13, 15].

SMRT sequencing uses hairpin adapters to attach to DNA fragments and create a single-stranded circular template that can be sequenced continuously. The sample is then loaded into a smart cell containing millions of zero-mode waveguides equipped with fluorescent nucleotides, such that each unique base is labeled with a unique color of fluorescent. Similar to nanopore sequencing, SMRT sequencing can distinguish modified bases from unmodified bases by measuring the time it takes to incorporate the next base during the DNA synthesis process as modified bases alter the kinetics of this process [13].

Previous studies have extensively evaluated the performance of different tools for methylation detection of nanopore sequencing [16, 17]. In this study, we present a systematic comparison of 5-mCpG methylation detection tools for nanopore sequencing (ONT) of 7179 DNA samples, including 22 samples sequenced with the latest nanopore flowcell chemistry, 132 oxBS sequenced samples from the same blood draw, and 50 samples sequenced using SMRT technology (PacBio). By analyzing large numbers of genomes, we accurately assess the reliability of CpG methylation predictions from nanopore long-read sequencing and introduce generalized quality filters that can be applied to other cohorts, providing guidance for researchers performing 5-mCpG studies based on long-read sequencing.

## Results

### Detection of CpG methylation with nanopore sequencing

We sequenced whole blood from 7179 individuals to an average coverage of 20.6 × per sample (median 19.5 × , ranging from 10 to 108 ×) on 8906 promethION flowcells from ONT. The same set of samples was used to investigate the correlation between CpG methylation, gene expression, and sequence variants (Stefansson OA, Sigurpalsdottir BD, Rognvaldsson S, Halldorsson GH, Juliusson K, Sveinbjornsson G et al: The correlation between CpG methylation and gene expession is driven by sequenced variance [Unpublished]). CpG methylation detection was performed using Nanopolish [18], which groups CpGs located within 10 bp of each other, referred to here as CpG units. Nanopolish takes reference-aligned reads as input and outputs for each read the strand of the reference that was sequenced and for each CpG unit a log-likelihood ratio (LLR) of it being methylated or not. The LLR is then translated to binary values indicating the methylation status of sequenced CpGs. We classified CpG units as “unreliable” when the LLR did not meet our criteria for predicting a CpG unit as either methylated or unmethylated. Here we restrict our analysis to 22,178,458 autosomal CpG units, containing the 27,651,488 CpG sites, detected by Nanopolish in our cohort.

### CpG methylation measurements are comparable between nanopore sequencing and oxBS

As a baseline for 5-mCpG rates, we used 132 DNA samples sequenced by oxBS in our previous study [19] to an average coverage of 25 × (median 24.7 × , range 15–41 ×). For each CpG unit, we calculated the average 5-mCpG rate over all individuals in each dataset separately (7179 in nanopore and 132 in oxBS) and assessed the performance of Nanopolish by evaluating the Pearson correlation coefficient between average 5-mCpG rates from oxBS data and the corresponding average 5-mCpG rates predicted from Nanopolish, across all CpGs. We refer to this correlation as per the CpG average Pearson correlation (APC).

Our analysis revealed a high APC between the 5-mCpG rates in the two datasets (*r* = 0.9594; 95%CI = 0.9594–0.9595) and the mean absolute difference (MAD) in the 5-mCpG predictions per CpG was 0.0471 (95%CI = 0.0471–0.0472) per CpG.

**Fig. 1:**
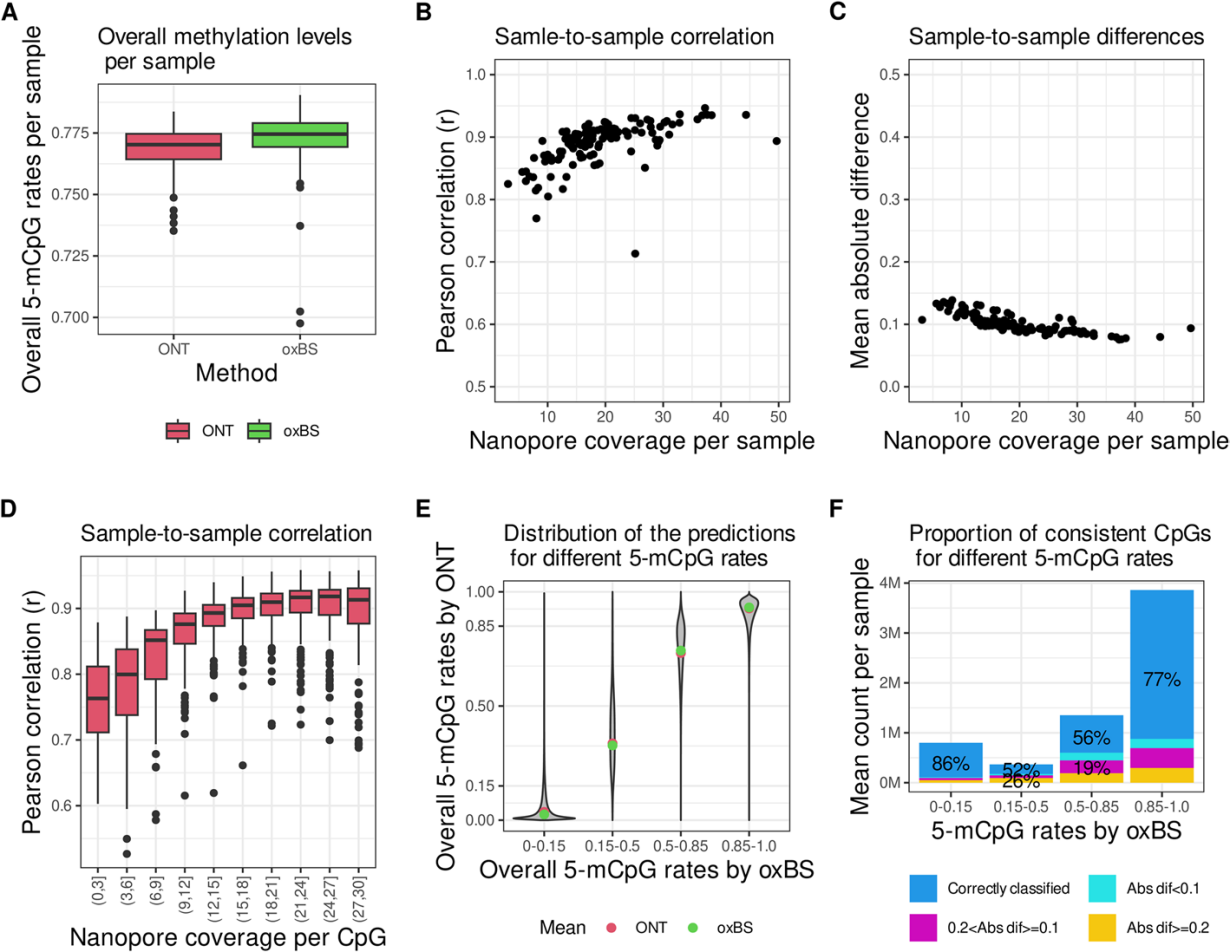
Nanopore sequencing and oxBS performance in the same DNA samples. The consistency in 5-mCpG rates measured by nanopore sequencing and oxBS in DNA samples isolated from the same 132 individuals was estimated by the following: **A** The overall measurement of 5-mCpG rates in each of the 132 DNA samples measured by ONT (red) and oxBS (green), *Y*-axis is limited to (0.7,0.8). The center line (solid black) shown in each box represents the median; the box limits represent the upper and lower quartiles; the whiskers represent 1.5 × interquartile range. **B** The Pearson *r* correlation coefficient, *y*-axis, and **C** mean of the absolute differences in 5-mCpG rates of each CpG, *y*-axis, with respect to nanopore sequencing coverage in each sample on the *x*-axis. Panels **D**, **E**, and **F** analyze sites that have > 25 × coverage in oxBS. **D** CpG coverage underlying the 5-mCpG rates, i.e., the number of sequences that were used to compute the 5-mCpG rate for a given CpG, in nanopore sequenced samples, *x*-axis, influences the consistency (Pearson *r*), *y*-axis, with 5-mCpG rates measured with high coverage by oxBS. The *y*-axis is limited to (0.5, 1) **E** CpG rates in nanopore (*y*-axis) and oxBS (*x*-axis, binned). The mean is represented with red (ONT) and green (oxBS). **F** Number (*y*-axis, unit = million CpGs) of correctly classified (blue) by nanopore sequencing in a sample-to-sample comparison. Incorrectly classified CpGs are colored according to the absolute difference in 5-mCpG rates (color legend)

We measured the overall methylation levels per individual by counting the number of times a methylated status was assigned to a CpG detected in sequences obtained from a given DNA sample to then divide this number by the total number of times we were able to assign a methylation status (unmethylated/methylated) to CpG sites in sequences obtained from that same DNA sample. We find that the overall methylation levels were on average lower in nanopore-sequenced samples than in those sequenced by oxBS ($\overline{x }$_Nanopolish_ = 0.767; 95%CI = 0.763–0.770 versus $\overline{x }$_ox-BS_ = 0.773; 95%CI = 0.770–0.775, Wilcoxon rank sum test *p* = 2 × 10^−6^) (Fig. 1A). As short-read sequences can be more difficult than long-read sequences to align to the reference genome, it is possible that these subtle differences in overall methylation levels between nanopore and oxBS sequenced samples are due to challenges in accurately aligning short-read sequences to the reference genome, which may affect the detectability and thereby measurement of certain CpGs by each of the two methods.

### Coverage affects the consistency of CpG methylation measurements in nanopore data

Next, we performed a matched sample-to-sample analysis based on the 132 individuals for which DNA samples were sequenced using both nanopore and oxBS and evaluated the Pearson correlation and MAD. We found that the correlation varied from 0.71 to 0.94 and the MAD from 0.076 to 0.14. The correlation was notably higher and MAD lower for high-coverage samples, indicating that sequencing coverage of approximately 12 × or more per sample is advisable for accurate methylation detection and sequencing at 20 × or greater yields even more accurate results (Fig. 1B, C). We then calculated the Pearson correlation for each sample, for all CpG sites with high sequence coverage (greater than 25 ×) supporting a minimum nanopore sequencing depth of a CpG unit as 20 × for obtaining a highly reliable measurement of its 5-mCpG rate (Fig. 1D).

The accuracy of the measured 5-mCpG rate is not affected by different versions of the basecalling algorithm nor changes in the error rate within the range of the reported error rate of nanopore sequencing (Additional file 1: Fig. S1, S2, Additional file 2: Tab. S1).

### Nanopore data is more consistent in unmethylated and methylated CpG units

To capture the distribution of the methylation predictions, we divided the paired data into four categories based on methylation rates in oxBS: unmethylated (0–0.15), low-methylated (0.15–0.5), intermethylated (0.5–0.85), and methylated (0.85–1). We found that Nanopolish predictions were consistent with oxBS measurements (Fig. 1E, Additional file 2: Tab. S2). We limit our analysis to CpGs with at least 25 × coverage in oxBS and consider a prediction made by Nanopolish to be correct if the prediction falls into the same of the four categories as the oxBS. We see that the highest fraction of correctly predicted CpG units was for unmethylated CpGs (86%), followed by methylated (77%), intermethylated (56%), and low methylated (52%) (Fig. 1F). The lower fraction of correct predictions among low- and intermethylated CpGs may be due to a higher propensity of the methylation in these categories to fall close to the boundaries of these classes and the higher variance of 5-mCpG rates expected for these categories, i.e., as the distribution of predicted methylation states is far more uniform for unmethylated and methylated CpGs in comparison to low- and intermethylated CpGs.

### Nanopolish methylation prediction quality is affected by CpG unit sequence context

Although the results of nanopore and oxBS are highly correlated, there are regions in the genome where methylation detection is more difficult due to limitations in the sequencing method, mapping, or methylation detection algorithms. To evaluate the performance of the methylation detection in nanopore-sequenced DNA, we compared the APC of CpG units located inside and outside of regions where we expected difficulties in methylation predictions.

**Fig. 2:**
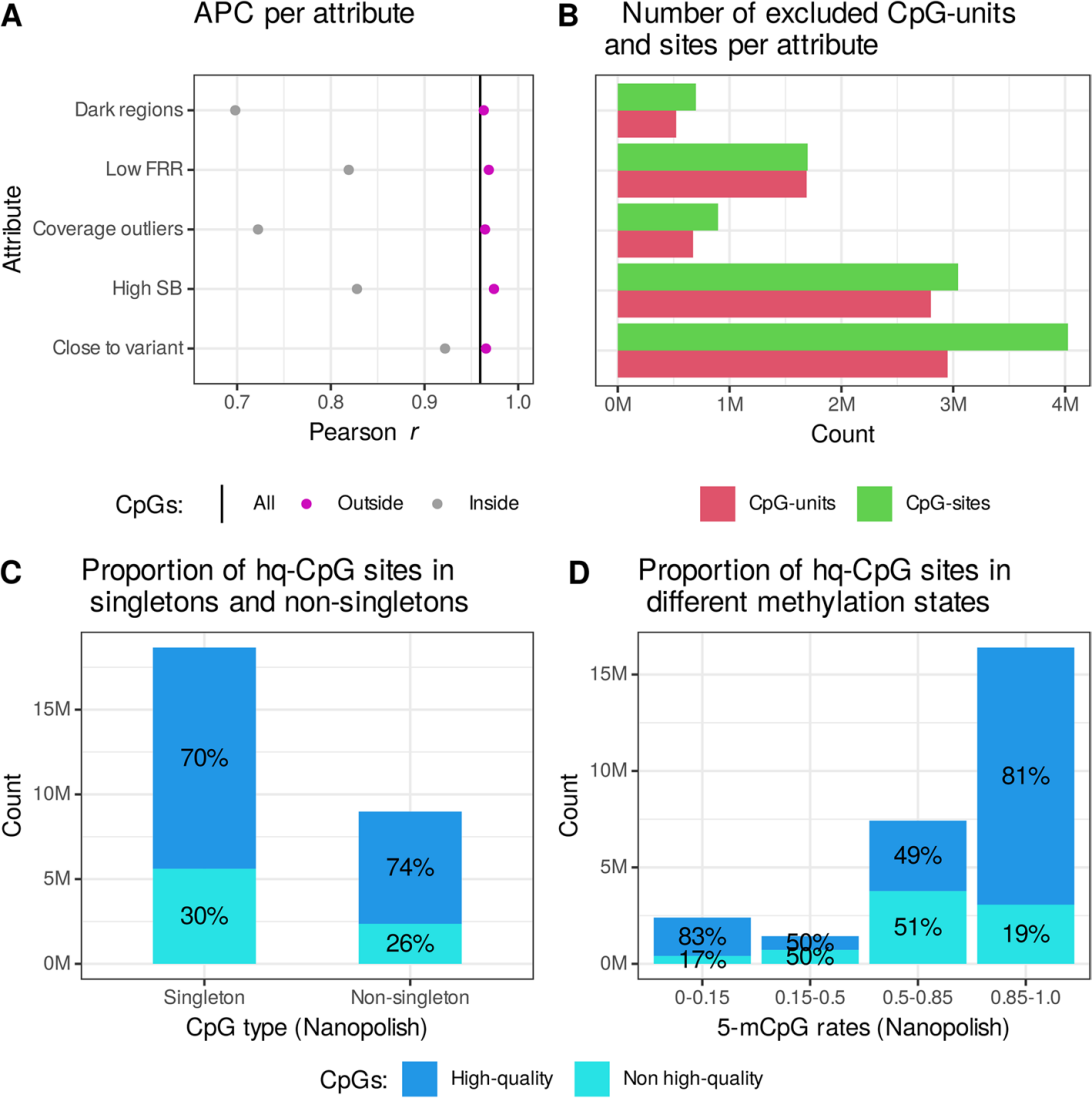
The quality of 5-mCpG rate measurements by DNA sequence attributes. **A** APC estimates (*x*-axis), for CpG sites located outside (pink) and inside (gray) of DNA sequence attributes, *y*-axis, and the APC estimates based on all CpGs (vertical black line). **B** The number of CpG units (red) and sites (green), *x*-axis, found inside of each attribute, *y*-axis. **C** The proportion of high-quality (dark blue) and non-high-quality (light blue) CpG units among singletons and non-singletons, *x*-axis. **D** The proportion of high-quality and non-high-quality CpG units within each methylation state category, *x*-axis, defined by binning the mean of 5-mCpG rates measured by Nanopolish

Nanopolish predicts methylation status from reads aligned to the human reference genome (GRCh38) [20], which instigates a risk of error when predicting the methylation status of CpG units located close to sequence variants. We found that CpG units located within 5 bp of a sequence variant had a lower APC (*r* = 0.9219; 95%CI = 0.9218–0.9221) than other CpG units (*r* = 0.96560, 95%CI = 0.96557–0.96563) (Fig. 2A). This likely is because Nanopolish assumes that aligned sequences are the same as those found in the reference genome. As a result, the electric signal, produced by a short stretch of a DNA sequence containing an unmethylated CpG, but including the alternative allele of a nearby sequence variant, may be similar to the signal produced in the presence of reference allele and a 5-mCpG.

We define dark regions [21] as sequences where ≥ 90% of the reads have mapping quality < 10, coverage < 5 × on average, and base quality < 20 in DNA samples analyzed on Illumina sequencers. Dark regions often contain large contiguous tandem repeats (e.g., centromeres and telomeres) or larger specific DNA regions that have been duplicated [21], causing the mapping to be unreliable. The APC for CpG units within dark regions was lower (*r* = 0.698; 95%CI = 0.697–0.699) than other CpG units (*r* = 0.96320; 95%CI = 0.96318–0.96323) (Fig. 2A). This poor correlation in these regions is likely largely attributable to the difficulty in measuring the methylation rates of CpG units that reside within these regions using oxBS, as mapping is generally more reliable in long reads. When the mapping is incorrect, the 5-mCpG rates are predicted from the wrong reference sequence leading to incorrect predictions.

We defined abnormal sequencing coverage, as greater than 1.5 times the average coverage or less than 0.5 times the average coverage, and show that these CpG units tend to have lower APC (*r* = 0.7223; 95%CI = 0.7218–0.7225) than other (*r* = 0.9646; 95%CI = 0.9645–0.9646) (Fig. 2A, Additional file 1: Fig. S3A, B), likely because of duplicated regions (such as tandem repeats) or mapping errors.

As DNA methylation is in most cases symmetric, meaning that cytosines in CpGs are methylated on both DNA strands [22], and hemi-methylated CpGs, where one strand is methylated while the other is unmethylated, are rare in the genome [23] we investigated strand bias, defined as the difference in the absolute value of the estimated 5-mCpG rates of the forward and reverse strands. We found that the magnitude of strand bias is low in oxBS data, with mean strand bias of 0.026 (quartiles = 0.0055, 0.028) (Additional file 1: Fig. S4). Strand bias was much higher in ONT Nanopolish data (mean = 0.095, quartiles = 0.017, 0.11, Wilcoxon rank sum test, *p* < 2 × 10^–16^), suggesting that strand bias may indicate problematic regions with unreliable methylation predictions. As there is far less strand bias in oxBS, we assume that these are unreliable in nanopore because of methylation detection artifacts. Notably, CpG units with strand bias greater than 0.2 (Additional file 1: Fig. S3C, D) had lower APC (*r* = 0.8279; 95%CI = 0.8275–0.8282) than other CpG units (*r* = 0.97411; 95%CI = 0.97409–0.97414) (Fig. 2A).

To further investigate the quality of methylation predictions in our nanopore-sequenced DNA samples, we examined CpG units with a low fraction of reliable reads (FRR), defined as the fraction of reads where the absolute log-likelihood ratio exceeds the defined cut-off. CpG units with FRR below 0.5 had a lower APC (*r* = 0.819; 95%CI = 0.816–0.820) than other CpG units (*r* = 0.96868; 95%CI = 0.96866–0.96871) (Fig. 2A, Additional file 1: Fig. S3E, F).

Consequently, we define problematic CpG units as being within dark regions, within 5-bp distance from a SNP, having coverage ≤ 0.5 times the average coverage or ≥ 1.5 times, strand bias ≥ 0.2, and FRR ≤ 0.5. These CpGs were removed from our analysis, resulting in a set of 15,644,462 (70.5%) high-quality CpG units (hq-CpGs), containing 19,685,181 (71.2%) CpG sites in the reference genomes (hg38). The APC for the hq-CpGs was 0.98582 (95%CI = 0.98581–0.98584) compared to 0.9594 (95%CI = 0.9594–0.9595) for the complete set and we found lowered MAD (Additional file 2: Tab. S4), between the predictions of hq-CpGs, indicating improved accuracy. The overall 5-mCpG rates were higher among hq-CpGs than among non-hq-CpGs (Additional file 2: Tab. S4). Furthermore, correlation coefficients were consistently higher for methylation measurements of hq-CpGs in the same DNA samples analyzed by Nanopolish and oxBS (Additional file 1: Fig. S5).

The highest number of CpG units were excluded from the set of hq-CpGs due to their proximity to a sequence variant, followed by high strand bias and low FRR (Fig. 2B). A similar proportion of singletons, defined as CpG units containing one CpG and non-singletons, were excluded from the set of high-quality CpG units or 30% and 26%, respectively (Fig. 2C). Notably, a higher proportion of low- (50%) and intermethylated (51%) CpG units were excluded from the set of hq-CpGs than unmethylated (17%) and methylated (19%) (Fig. 2D). Most CpGs (57.7%) are removed from the low- and intermethylated groups because of high strand bias. The hq-CpGs were evenly distributed across the number of CpGs within a unit and chromosomes (Additional file 1: Fig. S6).

### Guppy outperforms Nanopolish per CpG-site in comparison to oxidative bisulfite sequencing data

The recent improvements in algorithms for ONT basecalling have greatly enhanced the accuracy and efficiency of the basecalling. Specifically, a recent version of the basecaller, referred to as Guppy, can now perform CpG methylation detection at the basecalling stage by adding 5-mCpG to the DNA alphabet. We predicted the 5-mCpG rates of CpGs in 304 samples with Guppy (version 6.2.1) and calculated the average rates for each CpG over all individuals. Since Guppy does not group the CpGs like Nanopolish, we assumed the same rates for each CpG within a CpG unit in Nanopolish and compared the rates at the CpG site level.

The methylation calls from Guppy and Nanopolish were highly correlated, with an APC of 0.96558 (95%CI = 0.96555–0.96561) for the full set of CpGs. Guppy had higher APC with oxBS data (*r* = 0.97256; 95%CI = 0.97255–0.97259) than Nanopolish (*r* = 0.9594; 95%CI = 0.9594–0.9595). The overall 5-mCpG rates were lower for Guppy (${\overline{x} }_{Guppy}$ = 0.7634; 95%CI = 0.7633, 0.7635) than oxBS (${\overline{x} }_{oxBS}$ = 0.7756; 95%CI = 0.7755–0.7757; *p* < 2 × 10^−16^ Wilcoxon rank sum test). Interestingly, Guppy had lower mean strand bias ($\overline{x }$ = 0.064; quartiles = 0.016, 0.077) than Nanopolish ($\overline{x }$ = 0.095; quartiles = 0.017, 0.11; Wilcoxon rank sum test, *p* < 2 × 10^−16^), although the strand bias was still higher than in oxBS ($\overline{x }$ = 0.026; quartiles = 0.0055, 0.028; Wilcoxon rank sum test, *p* < 2 × 10^−16^).

By applying the same quality filters as specified for Nanopolish, we identified 22,256,402 (80.5%) hq-CpGs. This represents a 9.3% increase compared to the set of hq-CpGs identified using Nanopolish data. This difference is mainly explained by two factors: first this version of Guppy does not report number of reads where the probability of the call was below the threshold and therefore the FRR filter is not applicable, and second, Guppy has a lower strand bias, leading to more hq-CpGs being retained. The APC between the set of Guppy hq-CpGs and oxBS data was 0.98691 (95%CI = 0.98690–0.98693), compared to 0.97257 (95%CI = 0.97255–0.97259) for the complete set of CpGs (Additional file 2: Tab. S4, S5).

Moreover, we found high correlations between the matched samples for the methylation predictions generated by Nanopolish and Guppy, and Guppy and oxBS (Additional file 1: Fig. S7, S8). The sample-to-sample correlation between the 5-mCpG predictions from Guppy and the corresponding oxBS rates ranged from 0.62 to 0.90 for the full set of CpGs and increased to 0.65–0.91 for the set of hq-CpGs. For most samples, the correlation was higher between Guppy and oxBS than Nanopolish and oxBS (Additional file 1: Fig. S8A). The strand bias and MAD were also lower for Guppy on average per sample (Additional file 1: Fig. S8B, C).

### The latest chemistry attains higher accuracy and improved methylation predictions

ONT has made several improvements to its protein nanopore and motor protein, releasing nine versions of the system to date [15]. Our dataset consists mainly of samples sequenced on R9.4 flowcells (released in October 2016) and in addition we sequenced 22 samples on 28 R10.4 flowcells (received as early access) to an average depth of 9.64 × . R10.4 flowcells have two sensing regions designed to provide higher consensus accuracy with homopolymers than the R.9.4 flowcells [15].

The R10.4 flowcells have an average sequencing error rate [24] of 3.9%, significantly lower than the 8% average sequencing error rate for the R9.4 chemistry. Although there is high APC between 5-mCpG rates measured in all CpGs with the two types of flowcells (*r* = 0.98190, 95%CI = 0.98188–0.98191), the APC between 5-mCpG rates predicted from nanopore data in all CpGs and oxBS data is higher for R10.4 flowcells (*r*_R10.4_ = 0.97845; 95%CI = 0.97843–0.97846, *r*_R9.4_ = 0.97256; 95%CI = 0.97255–0.97259, Additional file 2: Tab. S5). R10.4 flowcells also show lower average strand bias of 0.047 (quartiles = 0.0097, 0.053) over all CpGs in comparison to R9.4 ($\overline{x }$ = 0.064; quartiles = 0.016, 0.077) (Wilcoxon rank sum test, *p* < 2e − 16) indicating improved accuracy (Additional file 2: Tab. S4). Nonetheless, the strand bias observed in R10.4 flowcells is still higher than that observed in oxBS data. Guppy R10.4 further showed lower MAD between methylation predictions with oxBS than Guppy R9.4 (Additional file 2: Tab. S4).

Applying the same quality filters as before to the R10.4 dataset, we obtain 22,893,522 (82.8%) high-quality autosomal CpGs, with APC of 0.99067 with oxBS (95%CI = 0.99066–0.99068, Additional file 2: Tab. S4, S5). This is a 2.3% increase in the number of hq-CpGs compared to Guppy data sequenced on R9.4 flowcells and an increase in APC.

### CpG methylation measurements are comparable between SMRT-sequencing, nanopore sequencing, and oxBS

We SMRT-sequenced whole-blood samples from 50 individuals on 170 flowcells to average sequencing coverage of 28.5 × per sample (range 13.6–41.7 ×), which was higher than for nanopore R9.4 and R10.4 sequencing methods (Additional file 1: Fig. S9A). The average N50, defined as the length of the sequence read at 50th percentile of the total sequence read length, was similar for SMRT and nanopore R9.4 and R10.4 sequencing methods (Additional file 1: Fig. S9B), but the average sequencing error rate was lower for SMRT-sequencing than either of the two nanopore sequencing methods, or 1.12% (range 1.02–1.31%, Additional file 1: Fig. S9C). We used primrose for methylation detection of SMRT-sequenced samples. The methylation detection step is performed by the sequencer after basecalling. The APC between predicted 5-mCpG rates across all 27,527,663 autosomal CpGs from SMRT-sequencing and oxBS data was 0.97010 (95%CI = 0.97008–0.97013) and the MAD was 0.05691 (95%CI = 0.05689–0.05694). After applying our quality filters, we identify 22,554,423 (81.9%) hq-CpGs of the autosomal CpGs with APC of 0.979956 (95%CI = 0.97955–0.97579) (Additional file 2: Tab. S4, S5). In summary, the number of hq-CpGs is similar to R10.4, with fewer filters applied and the APC with oxBS is lower than for either the R10.4 or R9.4 nanopore sequencing methods.

### Comparison of CpG methylation predictions from nanopore sequencing and SMRT sequencing

In this comparison, we used the 50 SMRT-sequenced samples (average coverage 26.7 ×) and 50 nanopore-sequenced samples analyzed using Nanopolish (average coverage 23.4 ×), 50 nanopore-sequenced samples on R9.4 flowcells and methylation called using Guppy (average coverage 22.0 ×), all of the 22 nanopore sequenced samples on R10.4 flowcells analyzed using Guppy (average coverage 9.64 ×), and 50 DNA samples sequenced by oxBS (average coverage 25.0 ×) (Additional file 2: Tab. S3).

We averaged the 5-mCpG rates over all samples and compared the APC correlation coefficient between all five methods (SMRT, R9.4-Guppy, R10.4-Guppy, R9.4-Nanopolish, and oxBS) and the absolute difference between 5-mCpG rates and oxBS (Table 1 (A)). 26,345,529 autosomal CpGs were detected in all datasets and used for the comparison. The highest APC was seen for Guppy applied to R10.4 and Guppy applied to R9.4. In comparison to oxBS, the highest APC and the lowest MAD were also seen for Guppy applied to R10.4 (Table 1 (A)). We note, however, that some of the differences in APC and MAD observed between methods may be due to differences in age, gender, or smoking status of the samples (Additional file 2: Tab. S3).

**Table 1:** Comparison between methods. (A) APC comparisons are shown below the main diagonal whereas MAD comparisons are shown above the main diagonal. (B) APC comparisons between methods based on all CpGs, or after restricting to those located close to sequence variants or those located within dark regions as indicated

**(A) Consistency between methods**										
			Mean absolute difference (MAD)							
			**OxBS**		**ONT Nanopolish**		**ONT Guppy R9.4**	**ONT Guppy R10.4**		**PacBio**
APC	OxBS		-		0.05933		0.05730	0.04772		0.05691
	ONT Nanopolish		0.9460		-		0.05040	0.04888		0.05207
	ONT guppy R9.4		0.9594		0.9653		-	0.04655		0.04608
	ONT guppy R10.4		0.9647		0.9637		0.9817	-		0.04363
	PacBio		0.9563		0.9571		0.9705	0.9785		-
**(B) Consistency between methods in sequencing context**										
	APC									
	**NP vs. Guppy R9.4**	**NP vs. Guppy R10.4**	**NP vs. PacBio**	**NP vs. oxBS**	**Guppy R9.4 vs. Guppy R10.4**	**Guppy R9.4 vs. PacBio**	**Guppy R9.4 vs. oxBS**	**Guppy R10.4 vs. oxBS**	**Guppy R10.4 vs. PacBio**	**PacBio vs. oxBS**
**All CpGs**	0.965	0.963	0.957	0.959	0.982	0.971	0.972	0.978	0.979	0.970
**CpGs near sequence variants**	0.930	0.921	0.913	0.922	0.973	0.957	0.940	0.934	0.962	0.929
**CpGs within dark regions**	0.841	0.845	0.841	0.698	0.906	0.873	0.716	0.736	0.876	0.691

Sequence variants around or within CpG introduce mapping bias in oxBS, leading to inaccurate methylation measurements and low APC. Therefore, it is less important to filter on CpGs located close to sequence variants for Guppy and PacBio, because low APC is most likely caused by inaccurate measurements in oxBS (Table 1 (B)) and higher APC is seen between Guppy R9.4, Guppy R10.4, and PacBio. We note however that likely all methods benefit from filtering on CpGs where sequence variants are located close to the CpG as all long-read sequencing technologies use the local sequence context and comparison to the reference genome for predicting the methylation status of CpGs. Not filtering on sequence variants would increase the number of hq-CpGs to about 25.1 M (90.7%) and 25.8 M (93.7%) hq-CpG for Guppy and PacBio with APC 0.98545 (95%CI = 0.98544–0.98546) and 0.97561 (95%CI = 0.97559–0.97563), respectively.

### Distribution of the 5-mCpG rates

**Fig. 3:**
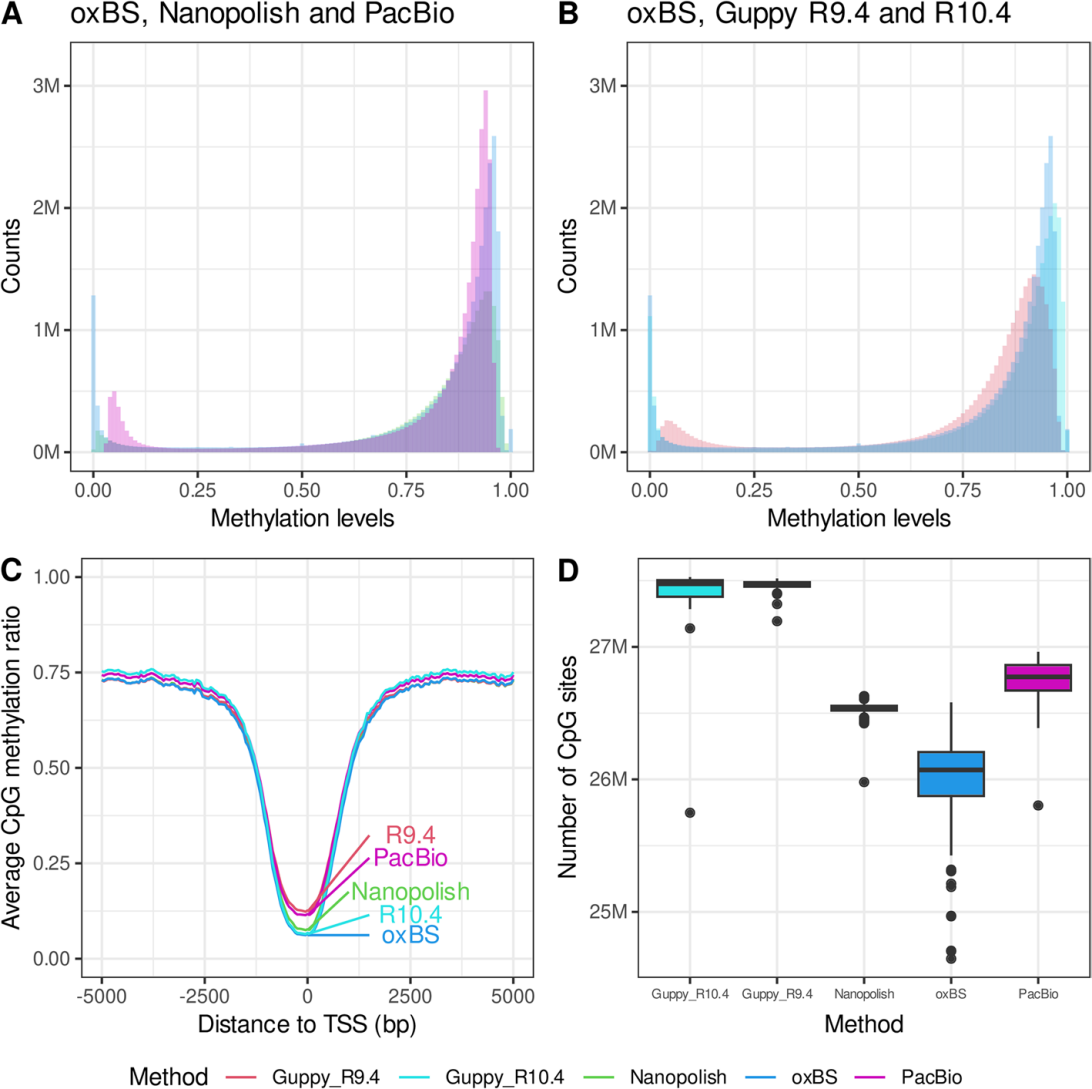
Comparison of CpG methylation detection by method. CpG methylation rates (ranging from 0 to 1) averaged across individuals yield the expected bimodal distribution seen in oxBS data for **A** oxBS, Guppy R9.4, and R10.4 and **B** oxBS, PacBio, and Nanopore. The units on *y*-axis are millions (M). **C** CpG methylation rates averaged in 50-bp bins relative to transcription start sites (TSSs) of genes expressed in whole blood. **D** Number of CpGs called by each method. For Nanopolish, we count all CpGs within a CpG unit. Note that the *y*-axis is limited from 24.5 to 27.7 M (millions). The center line (solid black) shown in each box represents the median; the box limits represent the upper and lower quartile; the whiskers represent the 1.5 × interquartile range

5-mCpG rates computed across all individuals in the five subsets of 50 individuals yielded the expected bimodal distribution for all methods (Fig. 3A, B). However, we noticed a shift in the distribution of methylated and unmethylated CpG sites away from 1 and 0, for both Guppy applied to R9.4 flowcells and PacBio. PacBio never reaches 0 or 1, while Guppy R9.4 rarely does. Guppy applied to R10.4 flowcells more closely follows the methylation distribution patterns seen in oxBS sequenced samples than R9.4. Additionally, all methods showed a higher number of intermethylated CpGs than oxBS. The distribution for hq-CpGs is similar with a slightly lower fraction of low- and intermethylated CpGs for Guppy R10.4 and PacBio (Additional file 1: Fig. S10). Less CpGs are removed due to strand bias and abnormal coverage for Guppy R10.4 and R9.4 compared to Nanopolish. Interestingly, more are removed because of abnormal coverage for PacBio (Additional file 1: Fig. S11).

### 5-mCpG rates of functional regions

To investigate the influence of biological context on the accuracy of the methylation predictions, we calculated the average 5-mCpG rates in 50-bp intervals relative to the start of the transcription start sites (TSSs) of genes expressed in whole blood. All methylation detection methods closely replicate the methylation patterns observed in oxBS-sequenced samples, which demonstrated a lack of methylation within TSSs (Fig. 3C). Notably, PacBio and Guppy R9.4 exhibited higher rates of CpG methylation at TSSs and lower rates away from TSSs, which is consistent with the slight shift in the methylation distributions observed for these two methods (Fig. 3A, B). Guppy applied to R10.4 flowcells, however, more closely follows the TSS methylation levels seen in oxBS (Fig. 3C). Further, Nanopolish has the lowest MAD with oxBS in unmethylated CpG units (Supplementary Fig. S12).

### Long-read sequencing calls more CpGs than oxBS

Long-read sequencing provides a significant advantage in the number of CpG sites captured over previous methods. To quantify this, we compared the number of CpGs called per sample by each long-read-based method and found that they all called similar number of CpGs. Restricting our analysis to autosomes, all three methylation detection tools for long-reads called similar number of CpGs (Guppy R9.4 = 27,467,383, Guppy R10.4 = 27,369,144, PacBio = 26,739,539 CpGs, and Nanopolish = 26,487,587, within 22,058,476 CpG units). As expected, oxBS called the fewest CpGs, with an average of 26,002,520 CpGs (Fig. 3D). The varying number of CpGs detected in long-read sequencing is most likely because of the criteria set by each method to make confident methylation predictions.

## Discussion

ONT and PacBio sequencing technologies both generate long reads but their underlying differences in chemistry affect the length of the reads, error rate, and throughput. Sequence detection algorithms can also affect the error rate. Consequently, each method has its distinct strengths and limitations. ONT excels in generating longer reads than PacBio, but this advantage comes at the expense of a higher error rate. Additionally, ONT is more scalable than PacBio resulting in lower sequencing cost per sample. Both of them exceed short-read sequencing in terms of capturing challenging regions, structural variants detection, read phasing accuracy, and creating whole chromosome assembly [13].

Long-read sequencing benefits from using native unamplified DNA for sequencing, because the molecules retain base modifications, allowing for their detection. This makes methylation detection more direct and simplifies the process. Several tools have been developed for methylation detection from long-read sequencing, such as Nanopolish [18], DeepSignal [25], DeepMod [26], Tombo [27], and Megalodon [28] for nanopore sequencing and ccsmeth [29] and modbamtools [30] for SMRT-sequencing. Extensive benchmark work on methylation detection tools for nanopore sequencing has been done previously [16, 17]. Liu et al. [17] concluded that Nanopolish and Guppy required the least amount of CPU time and exhibited the lowest peak memory usage, making it feasible for large-scale CpG methylation studies. Nanopolish and Guppy were also among the overall top performers, along with DeepSignal and Megalodon. However, Nanopolish and Guppy detected 4–6% fewer CpGs than DeepSignal and Megalodon, due to more stringent log-likelihood cutoffs [17]. These studies however were based on a small number of samples and did not consider the difference in methylation detection between sequencing methods or consider processing strategies to improve those correlations.

By using a large cohort, we can reduce the risk of drawing erroneous conclusions due to random variability and have more robustness to outliers. Our study extends beyond previous studies by showing that the quantification of methylation varies in quality between CpGs. We filter out unreliable CpGs and define a set of hq-CpGs that led to significantly improved accuracy while still providing a comprehensive analysis of over 70% of autosomal CpG sites. By tuning the quality attributes on a large cohort, they are more likely to be representative of the broader population and therefore generalized. The most significant improvement in APC was achieved by removing dark regions and regions with abnormal read coverage. APC between all pairs methods was lower for dark regions, suggesting that these regions are less reliable for all methods. Filtering out CpGs located ≤ 5 bp from sequence variants is necessary for Nanopolish because of the way the algorithm is designed, but this is not a necessary filtering criterion for other methods. All methods however likely benefit from filter on CpGs where a sequence variant occurs on either the cytosine or guanine base within the CpG motif itself. We note that the aforementioned filters may need to be reevaluated depending on each project’s needs.

We further show that Guppy applied to R10.4 flowcells with updated chemistry resolves some of the problems seen in the earlier versions of Guppy applied to R9.4 flowcells, such as strand bias, and results in a larger set of hq-CpGs. We report that for SMRT-sequencing the methylation predictions [12, 13] never reach either a fully unmethylated or fully methylated state. Changes to the model, such that the predictions do not regress away from either of these two extremes, may be beneficial.

The performance of long-read sequencing technologies relies heavily on the algorithms applied and the samples used as the training dataset. In many cases, the 5-mCpG detection algorithm is trained on fully methylated and fully unmethylated datasets, resulting in these regions being more accurately called than low- and intermethylated sites. For improved methylation predictions, penalized models, i.e., imposing additional cost on the models for making classification mistakes in these regions may improve the methylation detection. Furthermore, expanding the training dataset on more challenging regions and more human DNA sequences may improve the methylation predictions. Lastly, consensus approaches, based on the combination of predictions from two or more tools, show promising results for improved accuracy but were not investigated in this study [16].

CpG methylation detection from long-read sequencing faces limitation due to the diffusion of the signal around the CpG. Therefore, the algorithms require the use of intervals for the methylation detection and combination of the kinetic information from neighboring CpGs to increase the confidence in identifying methylated CpGs. Future work could identify problematic *k*-mers and incorporate that information into the training set to improve the detection reliability.

Long-read sequencing has revolutionized our ability to study CpG methylation without the need for chemical treatment of DNA, providing a higher resolution and more accurate picture of CpG methylation diversity. By enabling accurate phasing of the reads, long-read sequencing allows for precise characterization of DNA methylation at single base resolution at the haplotype level. This has facilitated the exploration of complex patterns of epigenetic modification and the detection of sequences that are infrequently depleted of methylation in the population that would have been missed using traditional array or short-read sequencing.

## Conclusions

CpG methylation detection in nanopore-sequenced DNA samples is highly accurate, even for samples with a high error rate and SMRT sequencing shows similar results. Based on our comparison, we made five key observations. First, coverage of approximately 10 × or higher per sample and per CpG is an important factor for accurate methylation detection. Second, we observed strand bias present in the nanopore data that is not seen in oxBS data. The strand bias decreases with a lower error rate and more accurate mapping and methylation predictions. Third, the methylation predictions from all methods are highly correlated and consistent with 5-mCpG detection in samples analyzed by the well-established oxBS method. They all replicate known 5-mCpG distributions in the human genome, such as the lack of 5-mCpG in promoter sequences. Fourth, we show improved consistency in 5-mCpG by excluding CpGs according to quality parameters identified herein. Between 7 and 30% of CpGs are filtered, depending on the dataset. The lower the error rate, and the more accurate the mapping of the sequenced DNA, the fewer CpGs need to be excluded for further analysis. Fifth, long-read sequencing detects about 3% more CpGs than oxBS. The number of CpGs detected by each long-read method mainly differs due to the criteria defined by each tool to confidently predict 5-mCpG rates. In summary, we have revealed the strengths and limitations of long-read sequencing methods, a crucial step to enable informed decision when selecting the appropriate sequencing technique and data analysis method.

## Methods

### Nanopore sequencing and analysis

#### Dataset

In this study, we sequence DNA isolated from whole blood samples from 7179 individuals (3745 females and 3434 males) participating in various studies at deCODE genetics. Analysis of structural variants in a subset of 3622 of these individuals has been described previously [19]. The earliest years of birth were 1890 and 1876, for females and males respectively, and the latest was 2015 for both genders. All individuals gave informed consent and all personal identifiers were encrypted by an external agent before being imported into the deCODE database.

#### Sample preparation

DNA from whole blood was extracted using the Chemagic method (perkinElmer), an automated procedure that involves the use of M-PVA magnetic beads. Sequencing libraries were generated using the SQK-LSK109 ligation kit from ONT. Sample input varied from 1 to 5 μg DNA, depending on the exact version of the preparation kit and the flowcell type used for the PromethION sequencing.

Samples were loaded onto PromethION R9.4.1 and R10.4.1 flowcells following ONT standard operating procedures. Sequencing was performed on PromethION devices.

#### Basecalling

The samples were analyzed with two versions of our pipeline, v3 (5761 R9 flowcells) and v4 (3145 R9 flowcells). The main difference between the pipelines is the version of the basecaller. In v3, squiggle data from PromethION was basecalled using Guppy 3.3.0 (3826 flowcells) using either the “flipflop” or “hac” model or 3.2.2 (536 flowcells), 3.6.0 (675 flowcells), and 4.0.14 (724 flowcells) using the “hac” model. In ont_build38_v4, all data was basecalled using guppy 5.0.11, using the “sup’ model (dna_r9.4.1_450bps_sup_prom.cfg). All 7179 individuals basecalled with guppy had a minimum reference-genome-aligned sequencing coverage of at least 10 × at the time of analysis and 3 × per flowcell.

#### Mapping

Basecalled reads were mapped to the human reference genome GRCh38 [20] with minimap2 [31], versions 2.14-r883 (5748 flowcells), 2.17-r941 (13 flowcells), and 2.22-r1105 (3145 flowcells). The aligned reads were sorted using samtools sort [32] and stored in a BAM file.

#### CpG methylation detection

All R9.4 flowcells were methylation called using Nanopolish [18] versions 0.11.1, 0.11.3, and 0.13.3. Nanopolish uses a hidden Markov model (HMM) to assign a log-likelihood ratio for the presence of cytosine methylation at each CpG site. We interpret values above 1.921 as indication for cytosine methylation and less than − 1.921 for unmodified CpG. Nanopolish groups CpGs within 10-bp distance and assigns a methylation status to each such that all CpGs within a group have the same methylation status. For this reason, we refer to CpGs measured by Nanopolish as CpG units. We first detect the methylation on the read level and exclude ambiguous methylation predictions (− 1.921 ≤ LLR ≤ 1.921). Then, we calculated the per unit methylation level by the fraction of reads classified as methylated out of all unambiguous reads.

The LLR threshold is selected based on Wilks’ theorem [33], which states that assuming the null hypothesis is true and the sample size approaches infinity, the distribution of the test statistics, − 2log(Λ), asymptotically approaches the chi-squared distribution with degrees of freedom equal to the difference in dimensionality. Here, Λ denotes the likelihood ratio. For 1 degree of freedom and *p*-value of 0.05, the chi-square value is 3.842. Therefore, we choose 1.921 as a threshold.

Additionally, we called CpG methylation in 304 samples on 325 flowcells using Guppy 5.0.11 or 6.2.1, which are versions of the basecalling algorithm that uses an extended alphabet, including 5mC. Guppy consists of a convolutional neural network (CNN) trained on fully methylated DNA created by treating the DNA with CpG methyltransferase M.SssI and fully unmethylated DNA created using PCR amplification. We then used the modBam2Bed (www.github.com/epi2me-labs/modbam2bed) script to extract the methylation values from the bam file and calculate the per-site methylation level.

#### Pipeline v3 vs pipeline v4

The main difference between the two versions is that v3 uses guppy versions 3.3.0, 3.2.2, 3.6.0, and 4.0.14 for the basecalling, resulting in an error rate of 11.53% on average, and v4 uses guppy 5.0.11 for the basecalling, resulting in an error rate of 8.06% on average (Additional file 1: Fig. S1). Version v3 is sequenced on older flowcells and hardware, potentially affecting the quality of the sequence reads and the methylation detection.

#### R10.4 flowcells

Additionally, we sequenced 22 samples on 26 R10.4 flowcells. Basecalling, alignment, and CpG methylation detection were performed on the box using Guppy 6.2.7. CpG methyl tags were then copied from the unaligned bam file to the aligned bam file and analyzed the same way as Guppy R9.4 samples.

### OxBS-sequencing and analysis

#### Dataset

All 132 samples were analyzed and described by Zink et al. [19].

#### Sample preparation

Samples were prepared using the TrueMethyl® Whole Genome kit (Cambridge Epigenetix) following the manufacturer’s recommendations (see URLs). In short, this involved a three-step procedure: (1) genomic DNA (0.2–0.4 μg) was oxidized using a proprietary oxidant (Cambridge Epigenetix). This step was done to convert all 5-hydroxy methylcytosines to their formyl derivatives, 5-formylcytosines; (2) bisulfite treatment of oxidized DNA converted both cytosines and 5-formylcytosines to uracil, leaving the 5-methylcytosines intact; (3) Illumina-compatible oxBS-seq libraries were prepared, using the appropriate primers and sequence adapters.

#### Sequencing

All sequencing libraries were quality control monitored for size and concentration using a LabChip GX analyzer (PerkinElmer). Libraries were first sequenced on a MiSeq system (2 × 25 cycles; Illumina) to evaluate quality (insert size, library diversity, etc.) and then underwent further WGS on either HiSeq 2500 system (2 × 125 cycles; Illumina) or HiSeq X system (2 × 150 cycles; Illumina) with ≥ 20% PhiX spike-in. The method was validated by sequencing four pairs of technical replicates and three pairs of matched biological replicates. Technical replicates were independent library preparations made from the same oxBS-treated DNA sample. Biological replicates were three pairs of samples from different individuals, matched on age, sex, and library quality parameters.

### SMRT sequencing and analysis

#### Dataset

We sequenced DNA isolated from whole blood samples from 50 individuals (29 females and 21 males) samples to an average depth of 26.73 × (range 12.74 × to 39.09 ×), on 189 flowcells. The earliest years of birth were 1941 and 1946, for females and males respectively, and the latest was 1998 for both genders.

#### PacBio sample preparation

Samples were prepared and sequenced using either protocol (A) (63 flowcells) or (B) (189 flowcells) as described below.

A) HiFi SMRTbell® prep kit 2.0

Genomic DNA (5 μg) diluted in Elution buffer (EB, 10 mM Tris, pH 8.5) was sheared to a target insert size of 15–20 kb using the MegaRuptor 3 system (Diagenode) with two successive shearing cycles at a speed setting of 31 and 32, respectively. Single-stranded overhangs were removed using the DNA prep enzyme master mix by incubating the reaction mixture at 37 °C for 15 min, followed immediately by incubation with the DNA Damage Repair mix v2 at 37 °C for 30 min. End-repair/A-tailing was done by incubating the reaction mix with the End Prep Mix for 10 min at 37 °C, followed by 65 °C for 30 min. Finally, adapter ligation using Overhang adaptor v3, ligation mix, ligation additive, and ligation enhancer was done by incubating the reaction mixture at 20 °C for at least 1 h. The resulting SMRTbell libraries were purified using AMPure® PB beads at a 1.0X volume (beads:sample) and eluted in 15 μL of EB. Damaged SMRTbell templates were removed by nuclease treatment using the SMRTbell Enzyme Clean Up Mix (15 μL sample/55 uL mix) by incubating the reactions at 37 °C for 30 min followed immediately by AMPure® purification as described above. Size selection of the HiFi SMRTbell libraries was performed using the Blue Pippin system (Sage Science). Approximately 1.5 μg of library in a final volume of 30 μL per sample was loaded on each lane of the system followed by 10 μL of loading buffer. Samples were run using the 0.75% DF Marker S1 High-Pass 6–10 kb vs2 Cassette definition file with a run time of 4.5 h and a selection mode of > 10 kb. The collected samples were purified using AMPure® PB beads at a 1.0X volume as described above and eluted in EB in a final volume of 11 μL. Purified SMRTbell libraries were quantified using the dsDNA HS assay kit on the Qubit fluorometer and assessed for sizing using the Fragment Analyzer 5300 (Agilent). Libraries were stored at − 20 °C until further use. All steps in the workflow were performed using wide-bore pipette tips and LoBind (Eppendorf) tubes and/or strips.

B) HiFi SMRTbell® prep kit 3.0

Genomic DNA (1 μg) was diluted in low TE buffer (10 mM Tris, pH 8.5, 0.1 mM EDTA) and sheared to a target insert length of 15–20 kb using the MegaRuptor 3 at a shear speed of 31. Samples were purified using a 1.0X volume ratio of SMRTbell clean-up beads and eluted in 47 μL of low TE buffer. Repair and A-tailing were performed in a mixture of End repair mix and DNA repair mix (RM1) in a reaction volume of 60 μL at 37 °C for 30 min, followed by 5 min at 65 °C. Adapter ligation was done by adding the RM2 mix (SMRTbell adapter, ligation mix, and ligation enhancer) to the samples in a final volume of 95 μL and incubating the mixture for 30 min at 20 °C, followed by 1X bead clean-up and elution in 40 μL of EB. Nuclease treatment was done using the RM3 mix by incubating the samples for 15 min at 37 °C. AMPure® PB bead size selection (< 5 kb) was performed by pre-diluting the beads to 35% (vol/vol) with EB and using a 3.1X (vol/vol) of diluted beads to each sample. Final elution was done in 15 μL of EB. Quantity and quality of purified SMRTbell libraries were done as described for method A.

#### PacBio sequencing

Run designs were created in the SMRT Link software (v 10 or 11). SMRTbell libraries were bound to Sequel II polymerase 2.2. using either the Binding Kit 2.2 or 3.2. Bound pol:DNA complex was purified using SMRTbell clean-up beads, quantified with Qubit, and loaded on the Sequel® II sequencing plate 2.0 with on-plate loading concentrations ranging from 30 to 70 pM, predictive loading enabled, and a maximum 2-h loading time. Samples were sequenced using the SMRT®Cell 8 M tray on the Sequel IIe system (HiFi application) with 30-h movie time per SMRT cell and kinetic data acquisition enabled. Each sample was in general sequenced on 3–5 SMRT® cells depending on HiFi yield.

#### CpG methylation detection

We use Primrose for methylation detection of SMRT-sequencing. During sequencing the kinetic information, pulse width and duration are stored for each CpG. The 5mC signature of the signal is quite diffused and not directly at the site of the modification but primarily at a few bases downstream. Therefore, SMRT sequencing uses the “aggregate on intervals” technique, where the kinetic information is combined for neighboring CpG sites, increasing the confidence in identifying the methylation at those sites [34]. For every CpG in a read, a feature vector is produced with the kinetics, pulse width and pulse duration for 16-bp intervals around each site on both strands. This feature vector is then fed into a convolutional neural network (CNN) that outputs the probability of methylation for each CpG per read.

The CNN was trained on modified native human DNA (HG002), where fully methylated DNA was generated by treating the DNA with CpG methyltransferase M.SssI, and fully unmethylated DNA was generated using whole genome amplification (WGA). The accuracy increases with the number of passes per read. The methylation probabilities for each CpG per read are stored in a methyl tag in a bam file. We then use the RefAlnBam-toModsBed-SAMTags.py script provided by PacBio to calculate the combined methylation per CpG and filter on minimum coverage 4 × .

### Statistical analysis

#### Per CpG average Pearson correlation (APC)

We calculate the average 5-mCpG rates per CpG or CpG unit over all individuals in the dataset. Then, we evaluate the Pearson correlation coefficient of the per CpG averaged methylation predictions to the corresponding averaged oxBS methylation rates.

#### Defining a set of high-quality CpG units

We assess the APC coefficient for CpGs that fall inside and outside any of the problematic regions, separately. We defined CpGs close to a variant as CpGs within 5 bp of any of 14,476,753 high-quality common variants. We define dark regions from 123 Illumina short-read sequenced samples, as regions where over 90% of the reads have mapping quality less than 10, coverage less than 5 × , and base quality less than 20 on average. We kept only regions at least 30 bp long. We define high-coverage regions as regions that have over 1.5 times the average coverage in the dataset and low-coverage regions as having less than 0.5 times the average coverage. We define strand bias as the difference in estimated 5-mCpG rates of forward and reverse strands. We further defined the fraction of reliable reads as a fraction of reads where the absolute log-likelihood ratio exceeds the defined cut-off as a fraction of the total number of available reads.

#### Statistical tests

Statistical tests were performed in R 3.6.0 [35]. Correlation and confidence intervals were calculated using the cor.test() function, and the statistical difference between the two distributions was evaluated using the non-parametric wilcox.test() function. Figures were created using ggplot2 [36].

### Supplementary Information

**Additional file 1.** Supplementary material: Supplementary notes, figures S1–S12, and data description.

**Additional file 2.** Supplementary tables: Supplementary Tables S1–S5.

**Additional file 3.** Review history.

## Data Availability

Access to the raw Icelandic sequence data is available on request from Kári Stefánsson at the premises of deCODE genetics. The data are not publicly available because of Icelandic state law. All analyses in the manuscript can be recreated using the 5-mCpG rates summary statistics, which can be downloaded from our website: www.decode.com/summarydata/, and from Zenodo [37].
