# Supplementary material for: A comparison of methods for detecting DNA methylation from long-read sequencing of human genomes
Source: Genome Biol. 2024 Mar 11;25:69. doi: 10.1186/s13059-024-03207-9 (PMC10929077; doi:10.1186/s13059-024-03207-9)
Supplement: Supplementary file 1 — Additional file 1. Supplementary material: Supplementary notes, figures S1–S12, and data description. [file 13059_2024_3207_MOESM1_ESM.docx]

# Additional file 1: Supplementary Material

**This document includes:**

1. **Supplementary Notes**
2. **Description of the contents in Data S1, S2, S3, S4, S5 and S6**
3. **References cited in Supplementary Materials**

# 1. Supplementary Notes

**1.1 Effect of different pipeline versions on the methylation calling**

All samples were analyzed with two version of our pipelines, referred to as v3 and v4. The main difference is the version of guppy, the basecalling algorithm, and flowcell chemistry, resulting in lower error rate for v4 (Fig. S1) (1). We show that the errror rate improves for each version of guppy (Fig. S2A). The sequencing coverage also increases which lowers the error rate (Fig. S2B) but is not affected by mean N50.


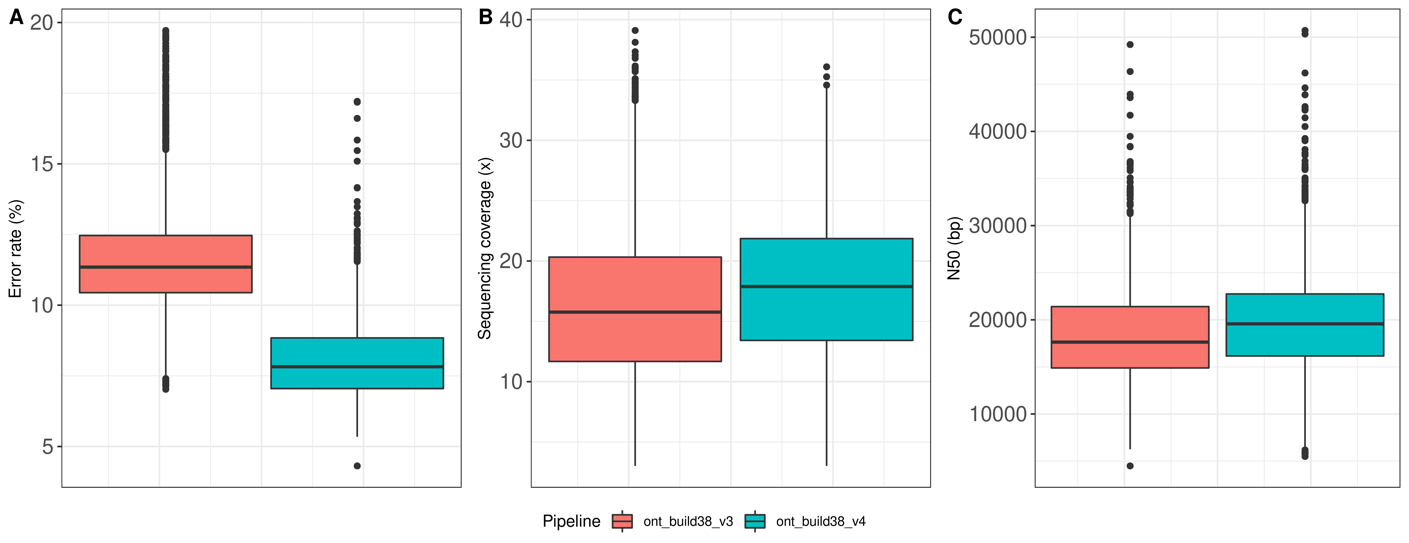
**Fig. S1** Statistics for different versions of the pipeline, computed for 50 high coverage samples. **A** Box plot showing the computed error rate for each version of the pipeline. **B** Box plot showing the sequencing coverage for each version of the pipeline. **C** Box plot showing the computed N50 for each version of the pipeline. The centre line (solid black) shown in each box represent the median; the box limits represent upper and lower quartile, whiskers represent 1.5x interquartile range.


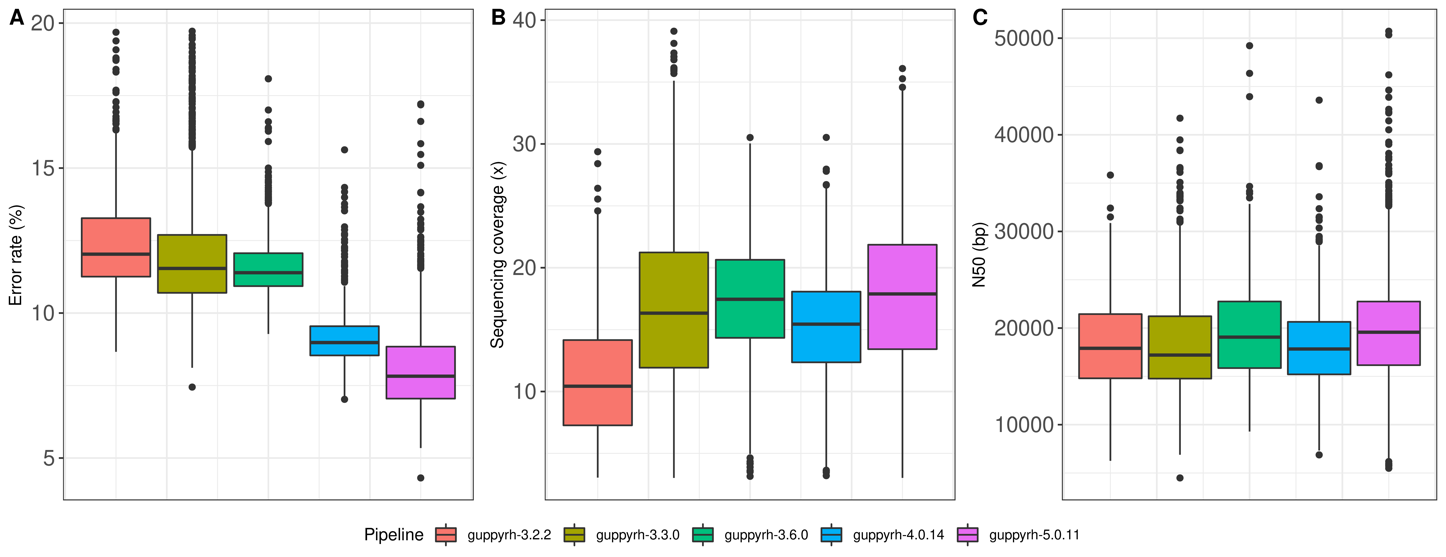


**Fig. S2** Statistics for different versions of Guppy, computed for 50 high coverage samples. **A** Box plot showing the computed error rate for each version of guppy. **B** Box plot showing the sequencing coverage for each version of guppy. **C** Box plot showing the computed N50 for each version of guppy. The centre line (solid black) shown in each box represent the median; the box limits represent upper and lower quartile, whiskers represent 1.5x interquartile range.

**1.2 Nanopore data is more consistent in unmethylated and methylated CpG-units**

CpGs were categorized based on 5-mCpG rates in oxBS, where sequencing coverage in oxBS is high (>25x). We calculated the mean methylation levels within correctly classified CpGs conditioned on the methylation levels measured on oxBS, separately for ONT and oxBS. We further calculated the mean absalute difference and standard deviation between the two measurements. Results are shown in Table S2.

**1.3 Nanopolish methylation prediction quality depends on the CpG-unit sequence context**


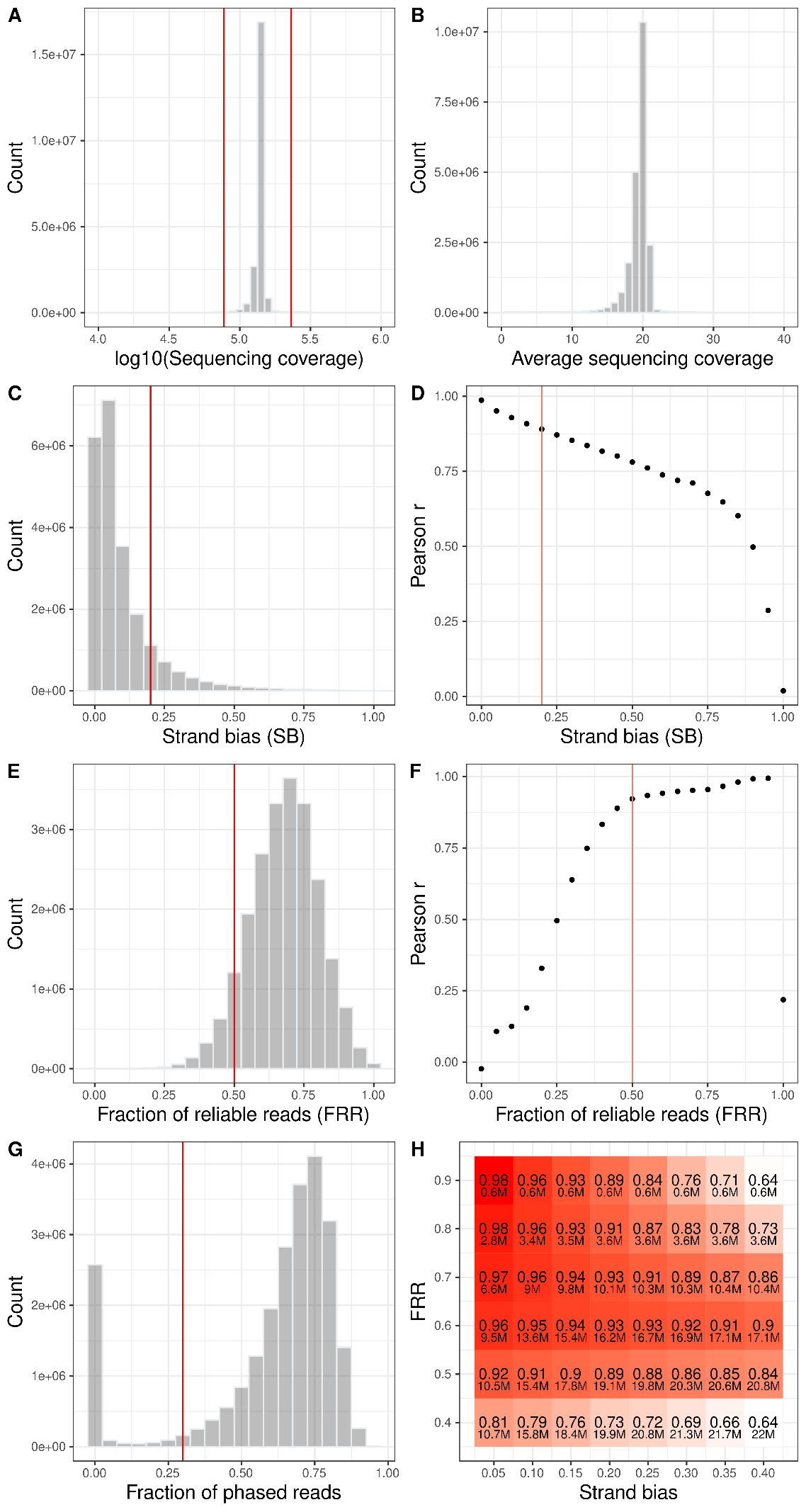


**Fig. S3** Selection of high-quality CpGs. **A** Histogram of the sequencing coverage distribution of the population, plotted on log10-scale. For better demonstration of the majority of the data we only show values between 4-6 on x-axis. **B** Histogram of the average coverage distribution (coverage divided by number of samples), shown for the range of 10-40x. **C** Histogram of the strand bias in the data. **D** Scatter plot of the correlation coefficient for each cut-off value for strand bias. **E** Histogram of the fraction of reliable reads. **F** Scatter plot of the correlation coefficient for each cut-off value for fraction of reliable reads. **G** Histogram of the fraction of phased reads. Red lines represent the selected cut-off values. **H** Heatmap of the correlation coefficient and number of hq-CpG based on each cutoff.

**
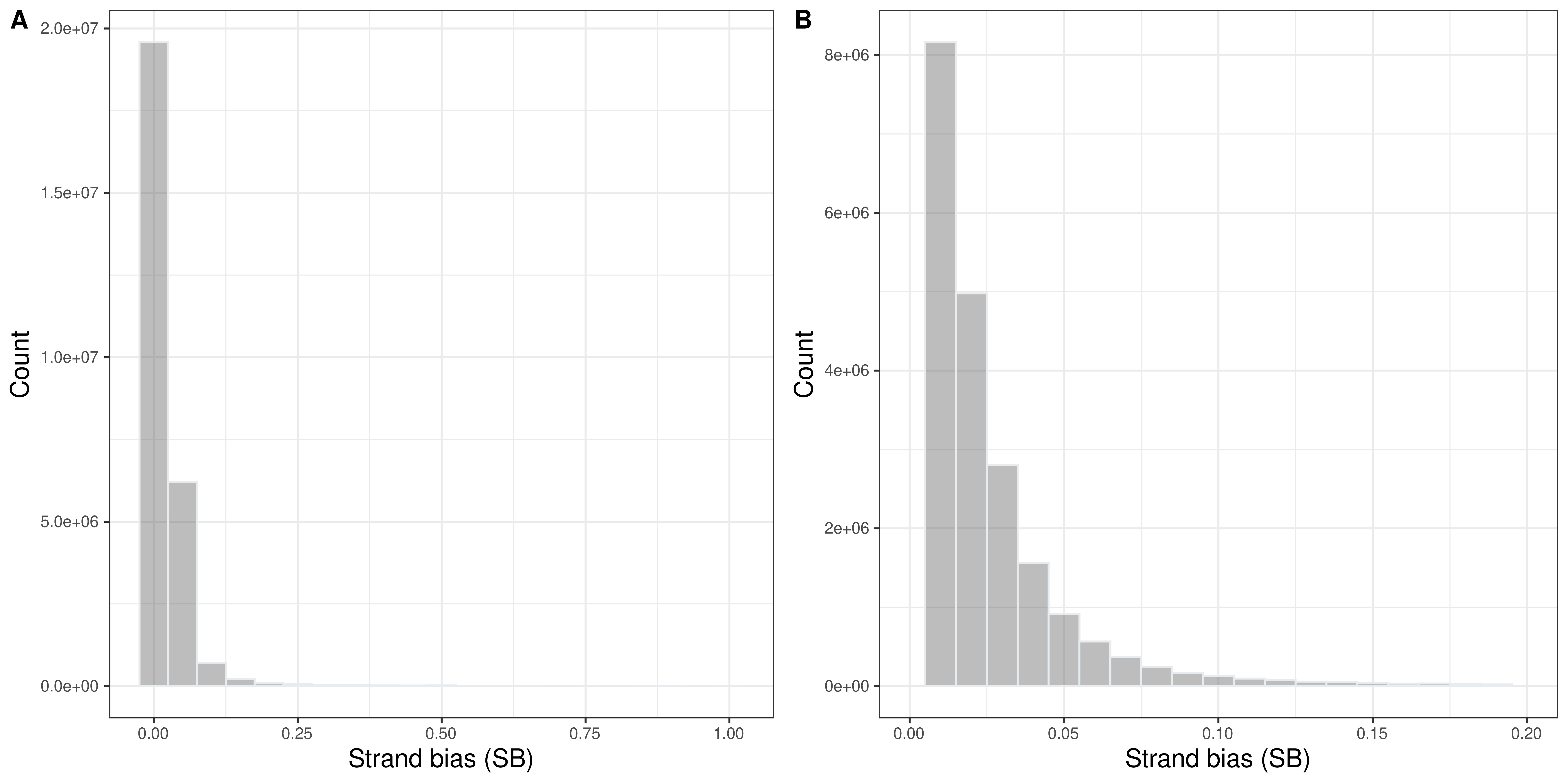
**

**Fig. S4** Strand bias in oxBS data. **A** Histogram for strand bias in oxBS data. **B** Same plot, shown only for strand bias, x-axis, between 0-0.2. The mean strand bias in oxBS data was 0.025.


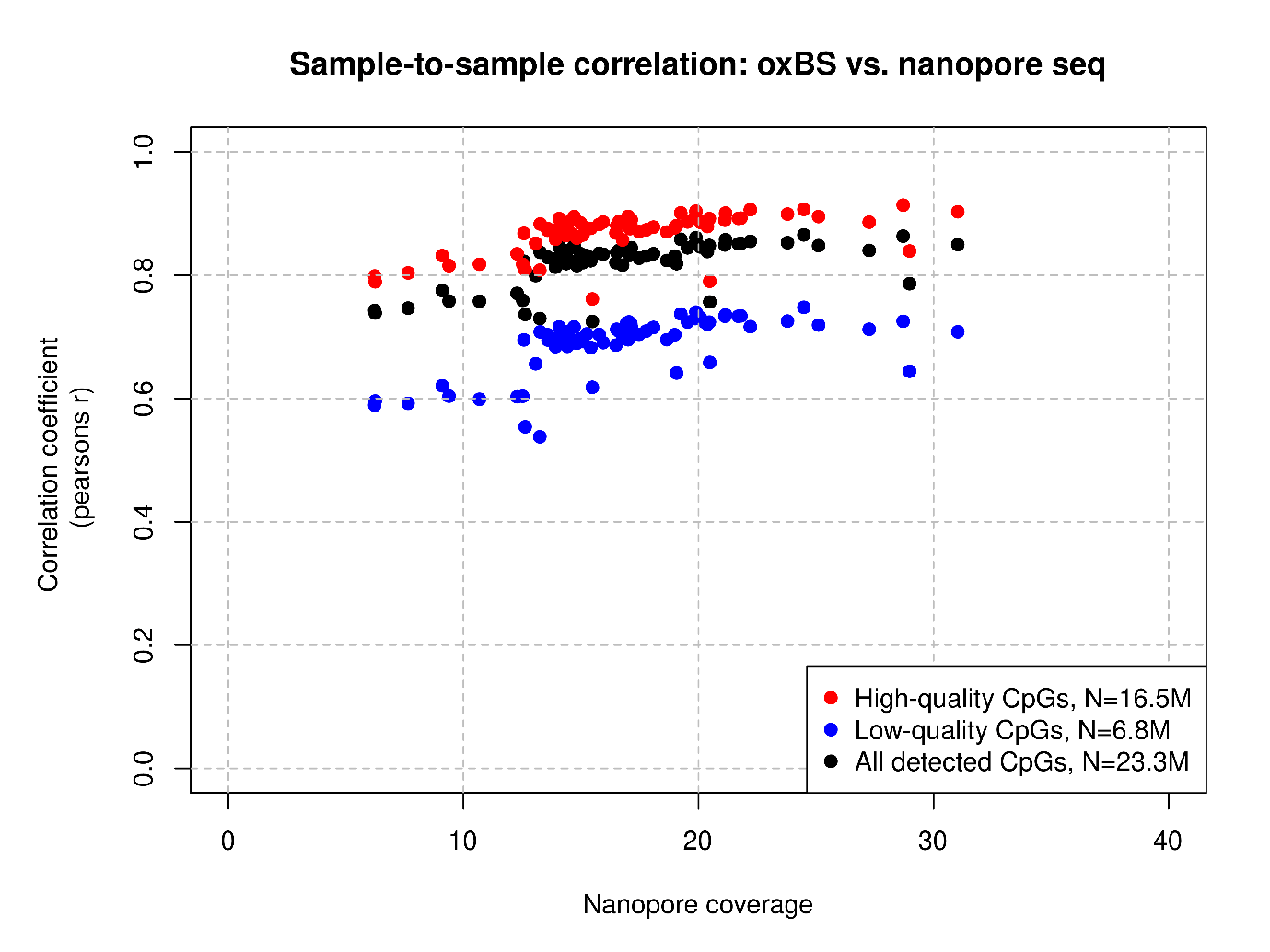


**Fig. S5 Sample-to-sample correlation for samples sequenced using oxBS and nanopore sequencing** 132 samples isolated from the same whole blood were analyzed using both oxidative bisulfite sequencing and whole genome nanopre sequencing. Shown are correlation coefficients, y-axis, for each sample-to-sample comparison; i.e. 5-mCpG rates measured by nanopore sequencing compared to 5-mCpG rates measured by oxBS in the same individual are plotted on the y-axis. The average coverage of the nanopore sequenced DNA (upper panel) and oxBS sequenced DNA (lower panel) are plotted on the x-axis. Correlation coefficients were calculated on the basis of all CpGs detected (blue colour) in sequenced reads from both sequencing methods, but also based only on CpGs that meet our quality critera for percent error and strand bias (black) and those that do not meet this criteria (red). The 16.5M CpGs categorized as “high quality” (black) consistently show higher sample-to-sample correlation coefficients, but the correlation is clearly affected by average coverage; i.e. nanopore sequenced samples with average coverage < 10 show lower correlation coefficients across all categories of CpGs.


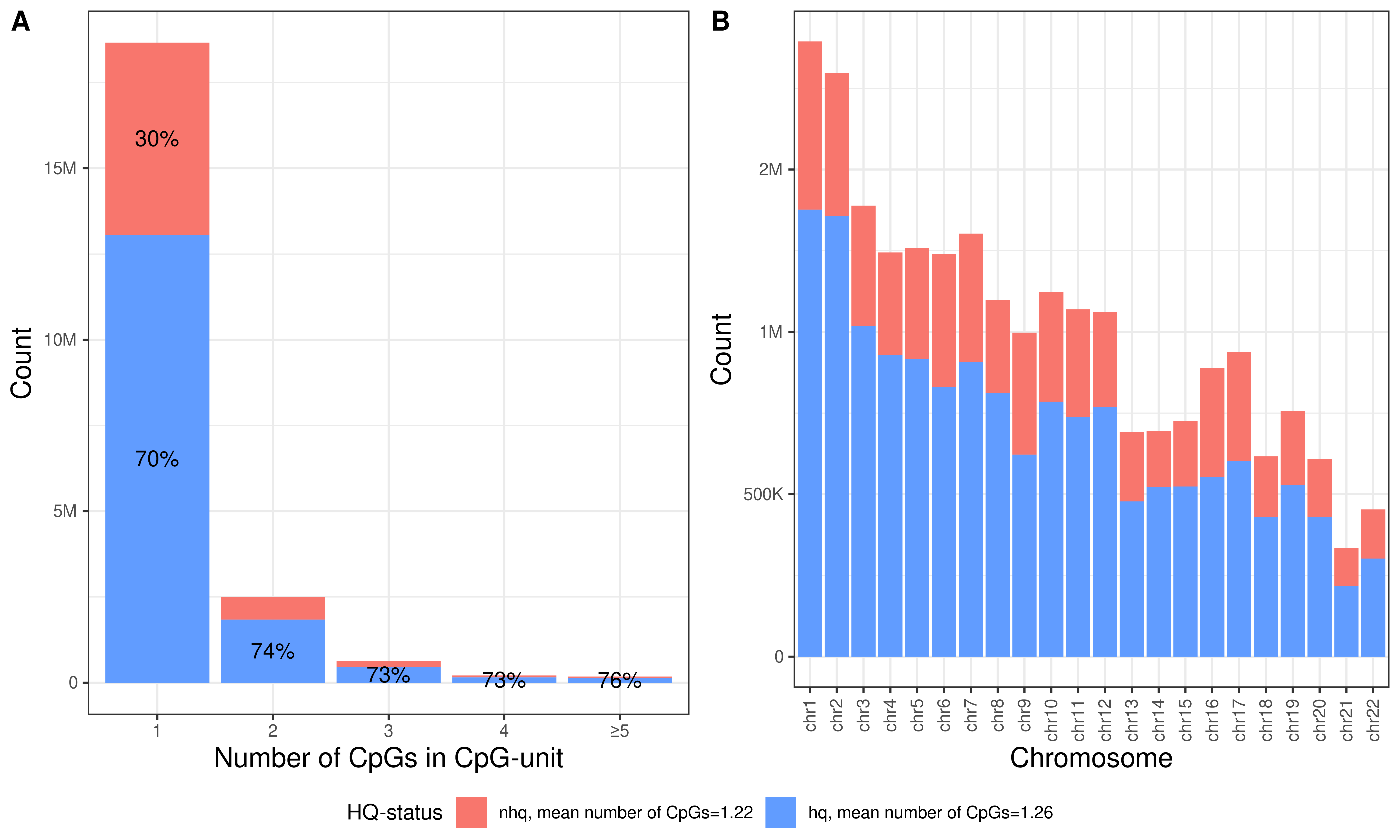


**Fig. S6** Distribution of high-quality CpG units**. A** Majority of the CpG units have less than 2 CpGs, for the full set and the hq-CpG units. The average number of CpGs within unit was 1.26 for hq-CpGs and 1.22 for other CpGs. **B** The distribution of hq-CpGs per chromosome is consistent with total number of CpGs per chromosome.

**1.4 Guppy outperforms Nanopolish per CpG site in comparison to oxBS**

We selected 42 samples, nanopore sequenced on 55 recent flowcells (average coverage 16.6x, average error rate 7.9%), for which we also have oxBS data. We performed additional methylation detection using Guppy. To evaluate the per-site correlation of methylation levels between the nanopore data and oxBS data, we select CpGs with high coverage in both datasets, defined as having on average more than 10 reads covering the CpG per flowcell for oxBS data and as having on average more than 10 reads covering the CpG, exceeding the cutoff threshold per flowcell for nanopore data. Using these high coverage CpGs, we create 10 benchmarking datasets with 0-10%, 10-20%, …, and 90-100% methylation levels in oxBS. We then sampled one CpG per 100kb in autosomes from each group, ending with 254,566 CpGs to compare (not all subsets per bin contained a high coverage CpG).

We examined the APC coefficient between the methylation levels predicted by the two tools and oxBS and found that Guppy has a higher correlation with oxBS than Nanopolish and has lower RMSE values (Fig. S7A). Nanopolish and Guppy agree with the expected methylation level per site for all groups and especially for intermethylated CpGs. Nanopolish is in slightly better agreement with oxBS for low methylated regions compared to Guppy (0-20%, m_Guppy_=0.180, m_Nanopolish_=0.151 and m_oxBS_=0.0905).

We then calculated the proportion of sites with concordant prediction within each window. We define concordant prediction as predictions where the methylation levels fall into the expected prediction window based on oxBS data. Nanopolish has more sites correctly predicted within expected window for unmethylated (0-10%, 10-20% and 20-30%) and highly methylated CpGs (90-100%) while Guppy has higher proportion correctly predicted in low- and intermethylated CpGs (30-90%) (Fig. S8B). Consistently, Guppy has higher number of CpGs within expected range for intermethylated CpGs (40-90%) while Nanopolish has higher number for unmethylated (0-20%) and highly methylated CpGs (90-100%). The performance is similar for low methylated CpGs (20-40%) (Fig. S7B).


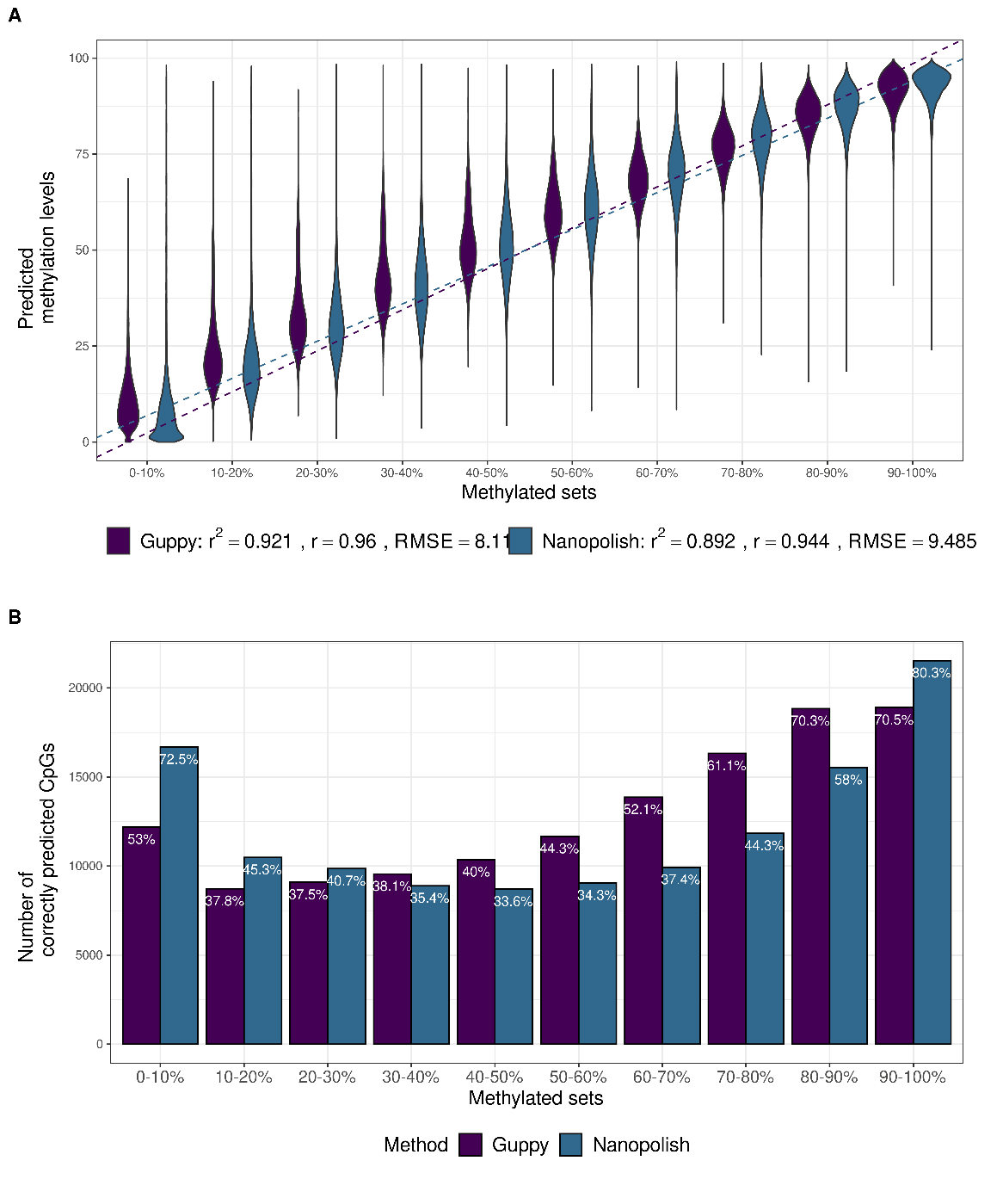


**Fig. S7** Comparison of CpG methylation predictions from Guppy and Nanopolish with oxBS within each methylation ratio group. **A** Violin plots showing the predicted methylation levels (y-axis) for each control set with a a given proportion of methylated reads (x-axis). The Pearson correlation coefficient, coefficient of determination and RMSE is given for both methods. **B** The number of congrugent methylation predictions (y-axis) and the proportion, shown for each methylation window (x-axis).

For each sample we calculated the Pearson correlation coefficeint over all CpGs between the predicted levels by Guppy or Nanopolish and the corresponding oxBS data. The per sample correlation ranged from 0.64 to 0.93, with average 0.85 (Fig. S8A). There was a similar average strand bias per sample in the two methods, with 0.162 on average per sample for Guppy and 0.167 for Nanopolish (Fig. S8B) and similar mean absolute difference between the two methods and oxBS.


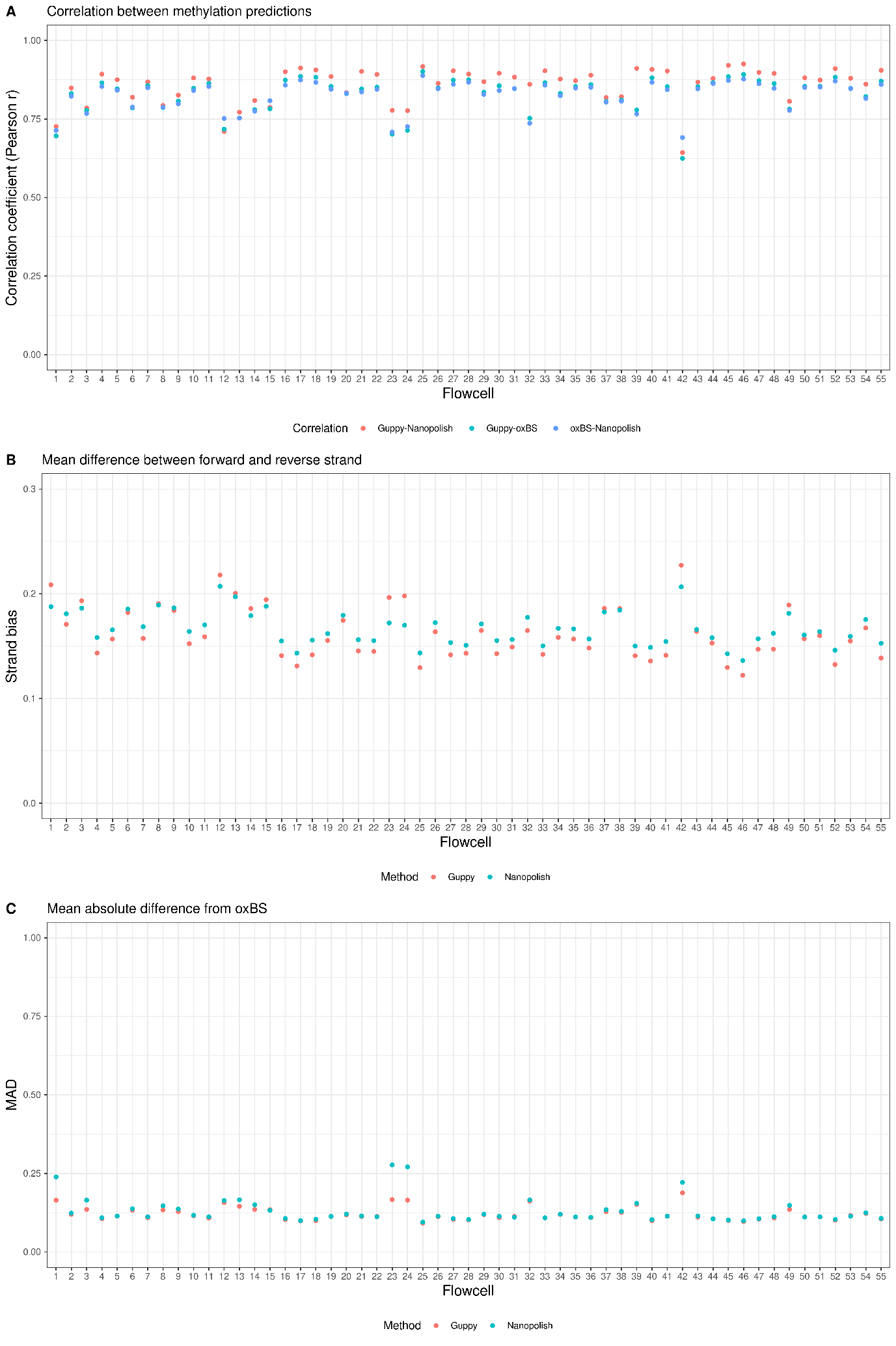


**Fig. S8** Comparison of CpG methylation predictions from Guppy and Nanopolish with oxBS. **A** Correlation coefficient (y-axis) per flowcell (x-axis). **B** Mean strand bias (y-axis) per flowcell (x-axis). **C** Mean absalute difference in 5-mCpG between Guppy and oxBS and Nanopolish and oxBS (y-axis) per sample (x-axis).

**1.5 Comparison of CpG methylation predictions from nanopore sequencing and SMRT sequencing**

Sequencing statistics for nanaopore and SMRT sequencing

**
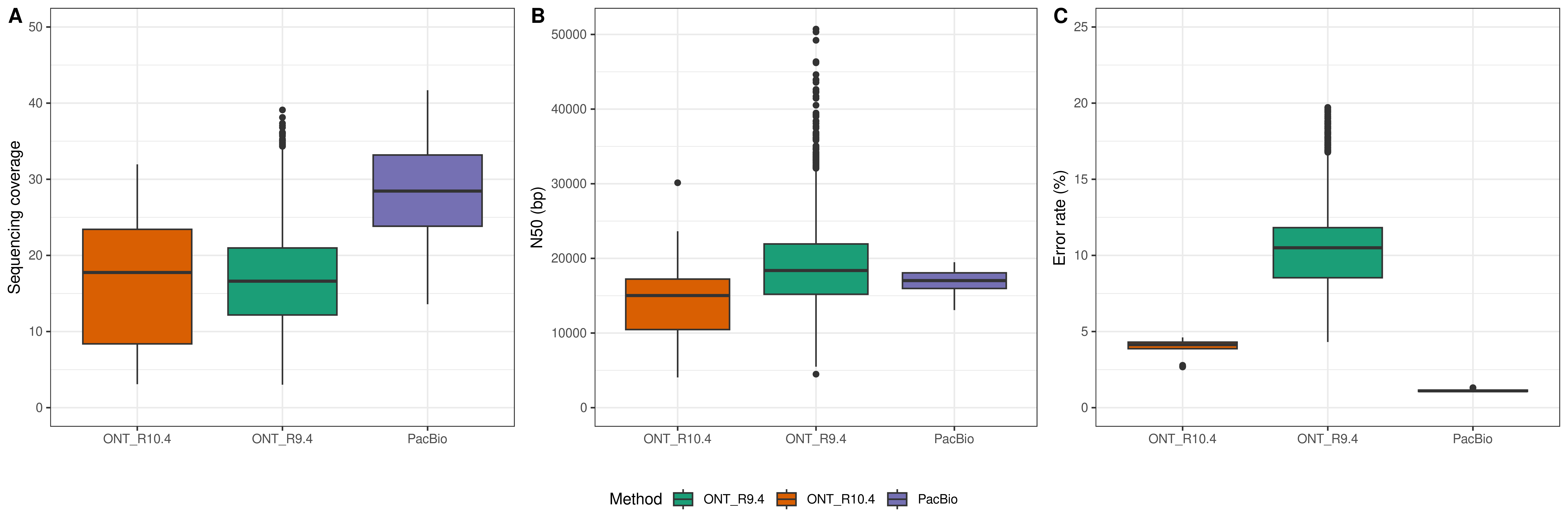
**

**Figure S9 Comparison of sequencing statistics for long-read sequencing techniques. A** Boxplot showing the sequencing coverage (y-axis) for each method (x-axis). **B** Box plot showing the N50 (y-axis) distribution for each method (x-axis). **C** Box plot showing the percentage error rate (y-axis) for each method (x-axis). The centre line (solid black) shown in each box represent the median; the box limits represent upper and lower quartile, whiskers represent 1.5x interquartile range.

Subset description

Description of the random subset created is shown in Table S3. We note, that some of the differences in APC and MAD observed between methods may be due to differences in age, gender or smoking status of the samples. The effect of these attributes involve relatively few and specific CpGs, considering the >15 million measured CpGs, and the effects tend to be subtle. For this reason, we assume that these attributes will not have a large effect when comparing all CpGs based on the APC parameter.

**Table S3** Description of the random subsets, showing gender and year of birth distribution. Smoking status was derived from ever smokers versus never smokers phenotype created inhouse.

| **Method** | **Gender** | **Number of samples** | **Earliest YOB** | **Latest YOB** | **Median YOB** | **Smokers** |
| --- | --- | --- | --- | --- | --- | --- |
| **oxBS** | Females | 26 | 1923 | 1989 | 1952 | 16 |
| **oxBS** | Males | 24 | 1931 | 1986 | 1951 | 14 |
| **Nanopolish** | Females | 28 | 2005 | 1914 | 1955 | 7 |
| **Nanopolish** | Males | 22 | 2001 | 1902 | 1972 | 6 |
| **Guppy_R9.4** | Females | 25 | 1991 | 1895 | 1952 | 12 |
| **Guppy_R9.4** | Males | 25 | 1998 | 1921 | 1955 | 6 |
| **Guppy_R10.4** | Females | 9 | 1992 | 1948 | 1979 | NA^*^ |
| **Guppy_R10.4** | Males | 11 | 2010 | 1975 | 1987 | NA^*^ |
| **PacBio** | Females | 29 | 1998 | 1941 | 1950 | 9 |
| **PacBio** | Males | 21 | 1998 | 1946 | 1949 | 9 |

^*None of the participants had smoking status available.^

Set of hq-CpGs

First, we assess if restricting to the set of hq-CpGs selected using Nanopolish methylation detection would benefit the Guppy data. Guppy benefit from restricting to the set of high-quality CpGs and we end up with similar correlation between Nanopolish and oxBS and Guppy and oxBS, however the Nanopolish data gains the most from the filters.

Next we applied the same filters for all methods. Not all filters are applicable for all methods, fraction of reliable reads is only filtered for Nanopolish and strand bias is not measured in PacBio as the reading strand information is not known. Fraction of reliable reads filters could be introduced for PacBio and Guppy, by slightly altering the summary scripts, but preliminary result suggest the benefit is not as great as for Nanopolish data. PacBio had higher APC in high and low coverage CpGs and more CpGs filtered out, indicating that this filter may need to be adjusted for PacBio data. Furthermore, PacBio showed the lowest improvement in APC of all methods, suggesting that other filters may be more beneficial for improving PacBio performance. In contrast, Nanopolish data required most filters, which explains why the number of hq-CpGs is lower in this dataset.

We found that applying quality filters improved the APC coefficient between long-read sequencing data and oxBS data. Nanopore data retained more CpGs in datasets sequenced using R10.4 flowcells and with more recent version of Guppy. Guppy applied to R10.4 flowcell and PacBio show higher fraction of high quality in all groups than Guppy applied to R9.4 (Fig. S10). After filtering, all datasets showed higher APC coefficient with oxBS data, lower MAD and higher methylation levels, indicating that more low- or unmethylated CpGs were removed. Guppy called on R10.4 flowcells had the highest number of hq-CpGs followed by PacBio and also highest APC with oxBS data and lowest MAD. R9.4 flowcells methylation called with Guppy and PacBio performed similarly (Additional File 2: Table S4, S5).


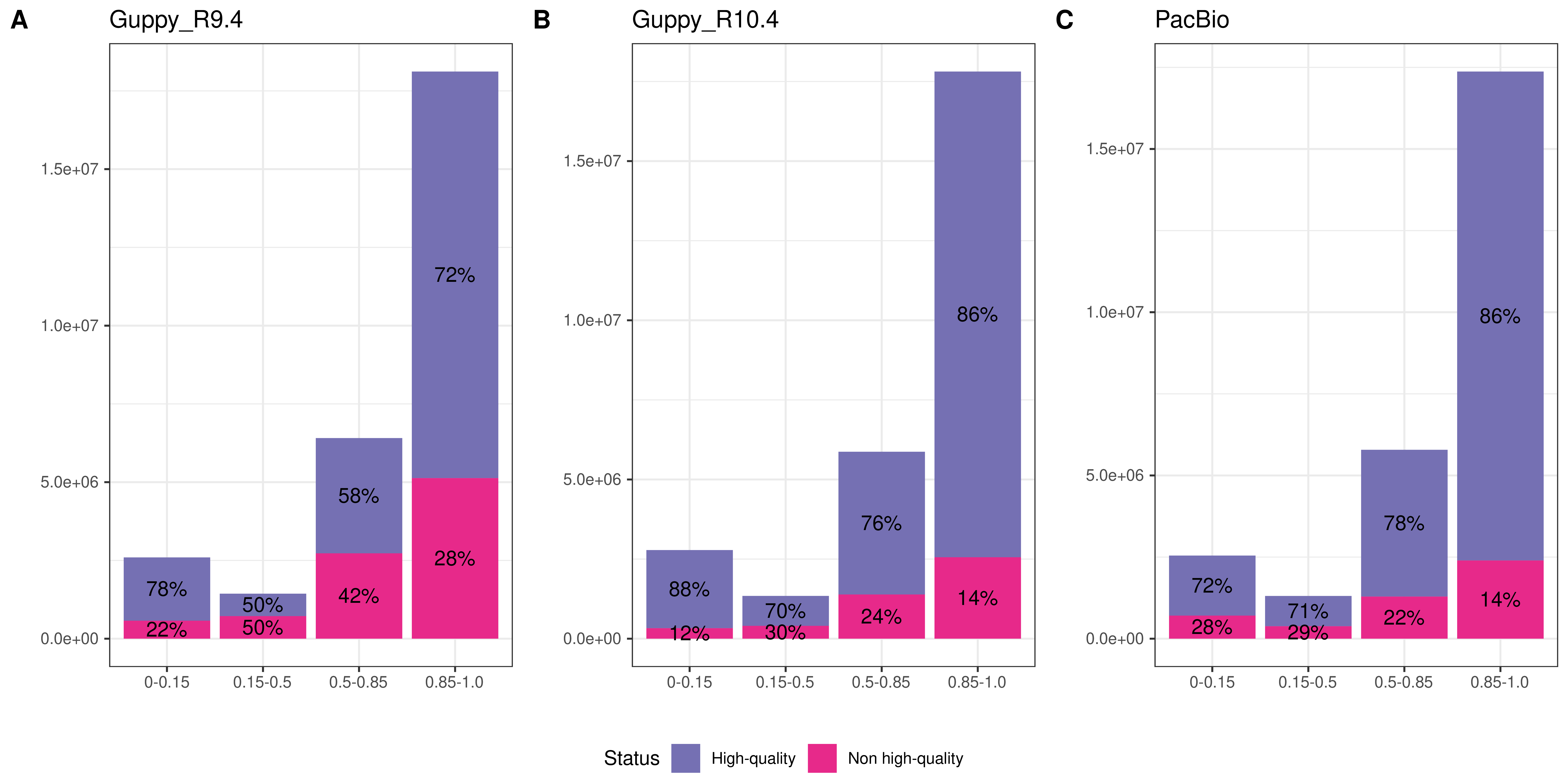


**Fig. S10** Fraction of hq-CpGs within each range in 5-mCpG rates, shown for **A** Guppy applied to R9.4 flowcells, **B** Guppy applied to R10.4 flowcells and **C** PacBio.


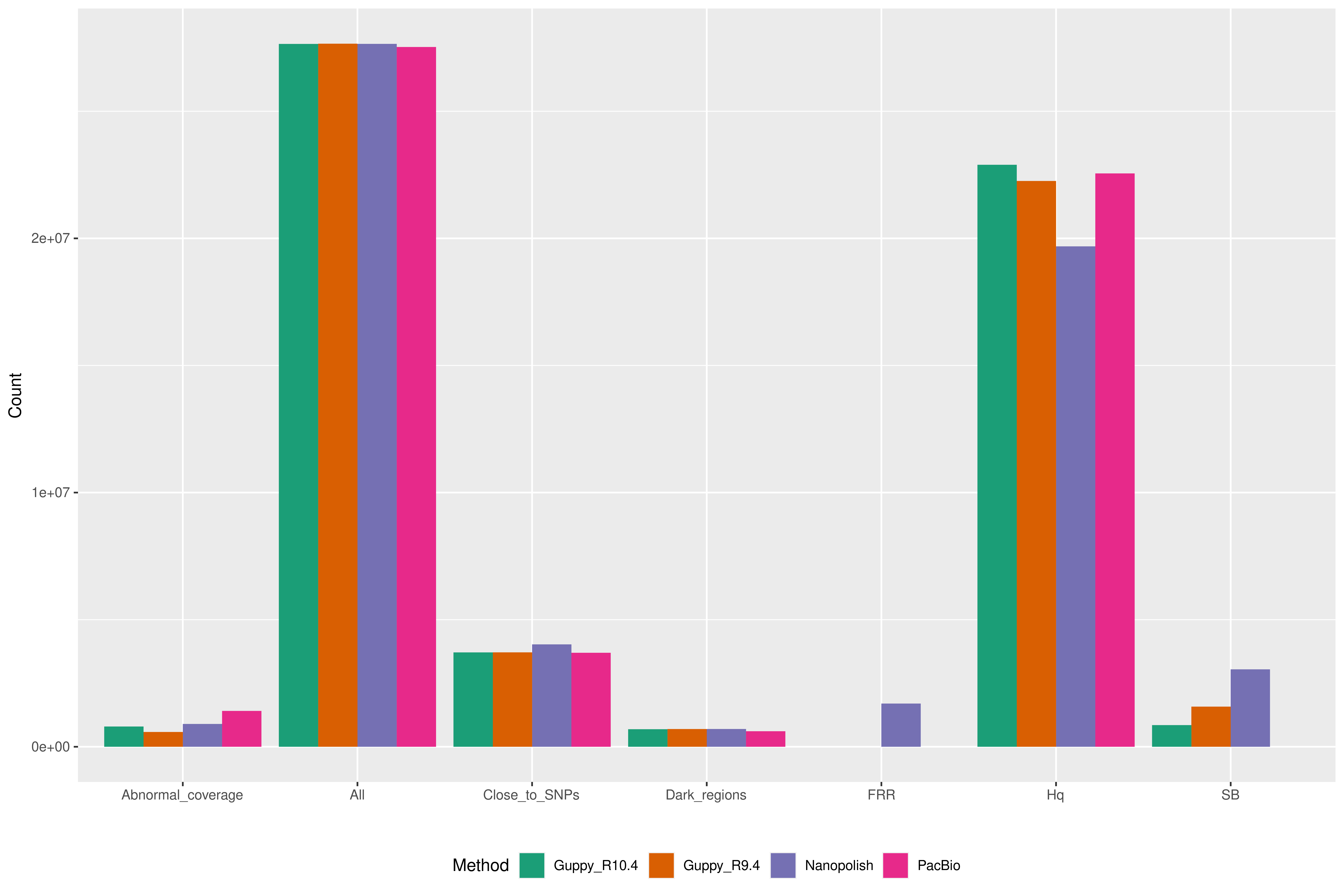


**Fig. S11** Number of CpGs (y-axis) removed from each group (x-axis) shown for Guppy R9.4 in orange, Guppy R10.4 in green, Nanopolish in purple and PacBio in pink. Strand bias is not measured in PacBio and FRR is only measured for Nanopolish.

Per-site accuracy of 5-mCpG predictions in different genomic and methylation context

To investigate the variation in methylation predictions among the different methods, we categorized the oxBS data into four bins based on the methylation rates and computed the MAD in 5m-CpG rates between all methods and oxBS. We further looked into the MAD for CpG islands, shelves and shores, separately.

In terms of the full set of CpGs and methylated CpGs all methods demonstrated similar performance with regards to MAD. All methods display a higher MAD in low- and intermethylated CpGs and all methods tend to overestimate the 5-mCpG rates compared to oxBS data, Guppy and PacBio more than Nanopolish, which supports the shift in bimodal distribution (Fig. 3., Additional File 1: Fig. S12). Nanopolish exhibits the lowest MAD in 5-mCpGs when compared to oxBS in unmethylated CpGs and CpG islands. On the other hand, PacBio has the lowest MAD in intermethylated CpGs. Adding more training data from inter- and lowmethylated CpGs might benefit the methylation detection of all tools.


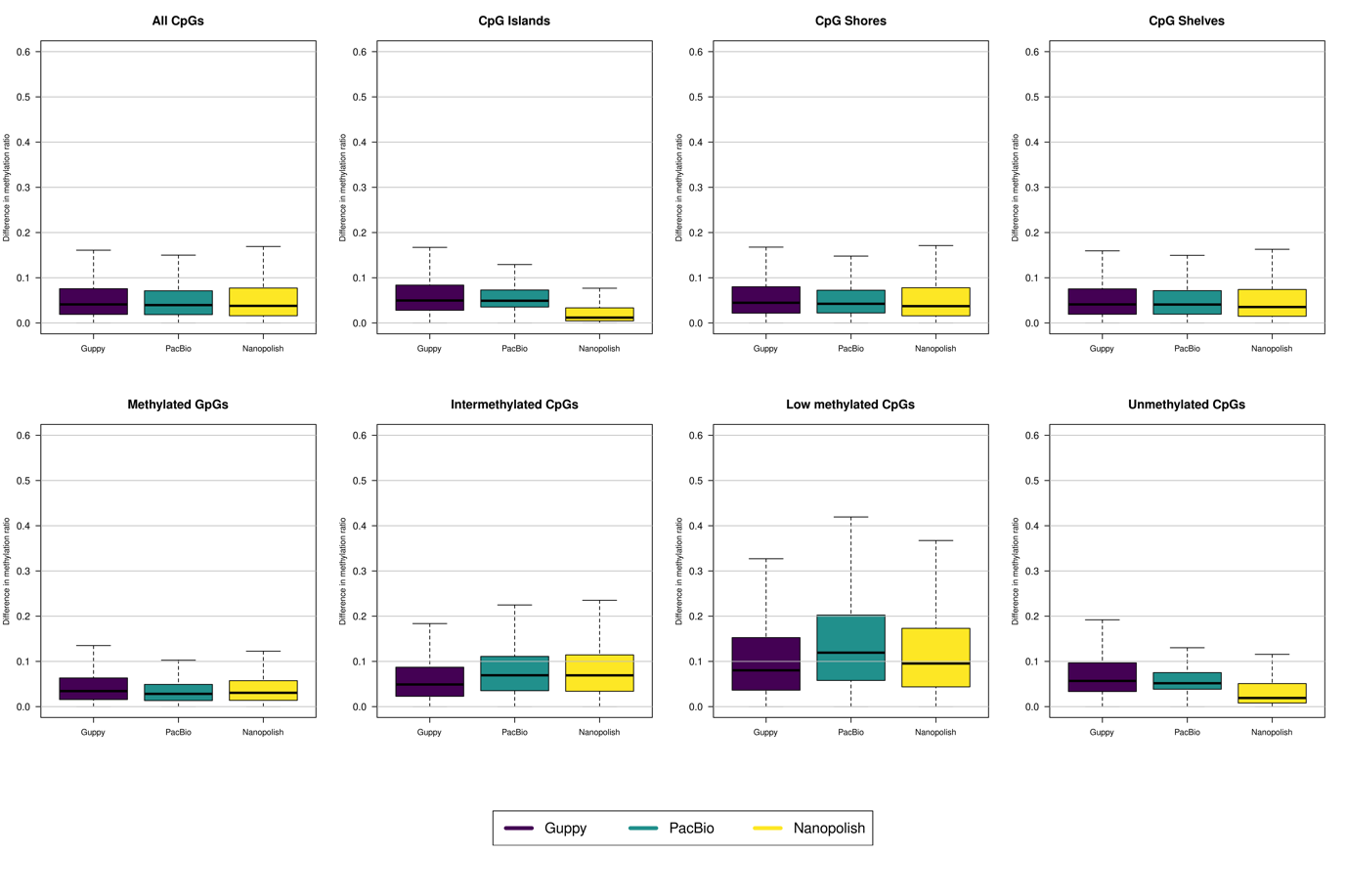


**Fig. S12** Mean absolute difference in 5-mCpG rates between the long-read datasets and oxBS data**.** Box plot showing MAD in 5-mCpG rates between all datasets shown separately for different genomic contexts: CpG islands, CpG shores and CpG shelves and for different methylation rates: methylated CpGs, intermethylated CpGs, low methylated CpGs and unmethylated CpGs. The centre line (solid black) shown in each box represent the median; the box limits represent upper and lower quartile, whiskers represent 1.5x interquartile range.

# 2. Supplementary data description

**Data S1:** Summary statistics for CpG units averaged over 7,179 samples sequenced using R9.4 flowcell from ONT and methylation called using Nanopolish.
***Chrom:*** *The chromosome of the CpG unit****start:*** *The nucleotide starting position of the CpG unit, 0 based****end:*** *The nucleotide ending position of the CpG unit****n_reliable:*** *The number of realiable reads behind the CpG unit. Reliable is defined as having the absalute log likelihood ratio larger than 1.921****n_total:*** *Total number of reads behind the CpG unit****ratio:*** *The methylation ratio, calculated as number of methylated reliable reads out of all reliable reads.****strand_bias:*** *Difference between the methylation ratio of forward and reverse strand****FRR:*** *Fraction of reliable reads, calculated as n_reliable/n_total****dark_region:*** *Boolean column indication of whether the CpG unit is within dark region. 1 indicates the CpG unit is within a dark region, 0 indicates it is not****SNP:*** *Boolean column indicating whether the CpG unit is within 5bp of SNP****phased_frac:*** *Fraction of phased reads****hq:*** *Boolean column Indicating whether the CpG unit is high-quality****hq_phased:*** *Boolean column indicating whether the CpG unit is high quality with phasing status included****highSB:*** *Boolean column indicating whether the CpG unit failes on strand bias test and has strand bias greater than 0.2****lowFRR:*** *Boolean column indicating if the CpG unit fails on FRR test and has FRR less than 0.5.****highCov:*** *Boolean column indicating if the CpG unit fails on coverage test and has coverage greater than 1.5 times the average coverage****lowCov:*** *Boolean column indicating if the CpG unit fails on coverage test and has coverage less than 0.5 times the average coverage****lowPF:*** *Boolean column indicating if the CpG unit fails on phasing fraction and has PF less than 0.3*

**Data S2:** Summary statistics for CpGs averaged over 132 samples sequenced using oxBS.
***Chrom:*** *The chromosome of the CpG****Pos:*** *The nucleotide starting position of the CpG, 0 based****N_total:*** *Total number of reads behind the CpG****ratio:*** *The methylation ratio, calculated as number of methylated reads out of all reads****Strand_bias:*** *Difference between the methylation ratio of forward and reverse strand*

**Data S3:** Summary statistics for CpGs averaged over 304 samples sequenced using R9.4 flowcell from ONT and methylation called using Guppy. ***Chrom:*** *The chromosome of CpG****startx:*** *The nucleotide starting position of the CpG, 0 based****end:*** *The nucleotide ending position of the CpG****n_total:*** *Total number of reads behind the CpG****ratio:*** *The methylation ratio, calculated as number of methylated reads out of all reads.****strand_bias:*** *Difference between the methylation ratio of forward and reverse strand****dark_region:*** *Boolean column indicating if the CpG is within dark region. 1 indicates the CpG unit is within a dark region, 0 indicates it is not****SNP:*** *Boolean column indicating if the CpG is within 5bp of SNP****highSB:*** *Boolean column indicating if the CpG failes on strand bias test and has strand bias greater than 0.2****highCov:*** *Boolean column indicating if the CpG fails on coverage test and has coverage greater than 1.5 times the average coverage****lowCov:*** *Boolean column indicating if the CpG fails on coverage test and has coverage less than 0.5 times the average coverage****hq:*** *Boolean column indicating if the CpG is high-quality*

**Data S4:** Summary statistics for CpGs, averaged over 22 samples sequenced using R10.4 flowcell from ONT and methylation called using Guppy.
***Chrom:*** *The chromosome of CpG****startx:*** *The nucleotide starting position of the CpG, 0 based****end:*** *The nucleotide ending position of the CpG****n_total:*** *Total number of reads behind the CpG****ratio:*** *The methylation ratio, calculated as number of methylated reads out of all reads.****strand_bias:*** *Difference between the methylation ratio of forward and reverse strand****dark_region:*** *Boolean column indicating if the CpG is within dark region. 1 indicates the CpG unit is within a dark region, 0 indicates it is not****SNP:*** *Boolean column indicating if the CpG is within 5bp of SNP****highSB:*** *Boolean column indicating if the CpG failes on strand bias test and has strand bias greater than 0.2****highCov:*** *Boolean column indicating if the CpG fails on coverage test and has coverage greater than 1.5 times the average coverage****lowCov:*** *Boolean column indicating if the CpG fails on coverage test and has coverage less than 0.5 times the average coverage****hq:*** *Boolean column indicating if the CpG is high-quality*

**Data S5:** Summary statistics for CpGs, averaged over 50 samples sequenced using SMRT-sequencing and methylation called using primrose.
***Chrom:*** *The chromosome of CpG****Pos:*** *The nucleotide position of the CpG, 0 based****n_total:*** *Total number of reads behind the CpG****ratio:*** *The methylation ratio, calculated as number of methylated reads out of all reads.****dark_region:*** *Boolean column indicating if the CpG is within dark region. 1 indicates the CpG unit is within a dark region, 0 indicates it is not****SNP:*** *Boolean column indicating if the CpG is within 5bp of SNP****highCov:*** *Boolean column indicating if the CpG fails on coverage test and has coverage greater than 1.5 times the average coverage****lowCov:*** *Boolean column indicating if the CpG fails on coverage test and has coverage less than 0.5 times the average coverage****hq:*** *Boolean column indicating if the CpG is high-quality*

**Data S6:** Summary statistics for CpGs, 132 samples sequenced using both oxBS and ONT, filtered on 25x per CpG in oxBS or greater.
***Chrom:*** *The chromosome of CpG****pos_ont:*** *The nucleotide start position of the CpG in ONT data, 0 based*

***end_ont:*** *The nucleotide end position of the CpG in ONT data, 0 based****ratio_ont:*** *The methylation ratio, calculated as number of methylated reads out of all reads in ONT.****pos_oxBS:*** *The nucleotide start position of the CpG in oxBS data, 0 based****ratio_oxBS:*** *The methylation ratio, calculated as number of methylated reads out of all reads in oxBS.*

# 3. References

1. Beyter D, Ingimundardottir H, Oddsson A, Eggertsson HP, Bjornsson E, Jonsson H, et al. Long-read sequencing of 3,622 Icelanders provides insight into the role of structural variants in human diseases and other traits. Nat Genet. 2021;53(6).
