# Supplementary material for: A comparison of methods for detecting DNA methylation from long-read sequencing of human genomes
Source: Genome Biol. 2024 Mar 11;25:69. doi: 10.1186/s13059-024-03207-9 (PMC10929077; doi:10.1186/s13059-024-03207-9)
Supplement: Supplementary file 2 — Additional file 2. Supplementary tables: Supplementary Tables S1–S5. [file 13059_2024_3207_MOESM2_ESM.docx]

# Additional file 2: Supplementary Tables

**Table S1** The mean absolute difference between Nanopolish methylation predictions and the corresponding oxBS methylation levels.

|  | **Mean absolute difference** | **Standard deviation** | **Pearson r** |
| --- | --- | --- | --- |
| **Pipeline v3 (AER=12.3%)** | 0.0494 | 0.690 | 0.955 |
| **Pipeline v4 (AER=7.94%)** | 0.0486 | 0.0694 | 0.954 |

**Table S2** Mean methylation levels for oxBS and nanopore within correctly classified CpGs, mean absalute difference and standard deviation between nanopore methylation detection and oxBS, conditioned on methylation levels measured in oxBS.

| Average methylation in oxBS | **Mean methylation levels (oxBS)** | **Mean methylation levels (ONT)** | **Mean absolute difference** | **Standard deviation** |
| --- | --- | --- | --- | --- |
| **Unmethylated (0-0.15)** | 0.0362 | 0.019 | 0.0226 | 0.0247 |
| **Low methylated (0.15-0.5)** | 0.336 | 0.307 | 0.0673 | 0.0560 |
| **Intermethylated (0.5-0.85)** | 0.732 | 0.731 | 0.0588 | 0.0526 |
| **Methylated (0.85-1)** | 0.927 | 0.936 | 0.0266 | 0.0228 |

**Table S3** Description of the random subsets, showing gender and year of birth distribution. Smoking status was derived from ever smokers versus never smokers phenotype created inhouse.

| **Method** | **Gender** | **Number of samples** | **Earliest YOB** | **Latest YOB** | **Median YOB** | **Smokers** |
| --- | --- | --- | --- | --- | --- | --- |
| **oxBS** | Females | 26 | 1923 | 1989 | 1952 | 16 |
| **oxBS** | Males | 24 | 1931 | 1986 | 1951 | 14 |
| **Nanopolish** | Females | 28 | 2005 | 1914 | 1955 | 7 |
| **Nanopolish** | Males | 22 | 2001 | 1902 | 1972 | 6 |
| **Guppy_R9.4** | Females | 25 | 1991 | 1895 | 1952 | 12 |
| **Guppy_R9.4** | Males | 25 | 1998 | 1921 | 1955 | 6 |
| **Guppy_R10.4** | Females | 9 | 1992 | 1948 | 1979 | NA^*^ |
| **Guppy_R10.4** | Males | 11 | 2010 | 1975 | 1987 | NA^*^ |
| **PacBio** | Females | 29 | 1998 | 1941 | 1950 | 9 |
| **PacBio** | Males | 21 | 1998 | 1946 | 1949 | 9 |

^*None of the participants had smoking status available.^

**Table S4 Statistics for the full set of CpGs compared to hq and not hq-CpGs.** Mean methylation, APC coefficient, MAD, strand bias and number of CpGs shown for each method. Strand bias is not shown for PacBio as both strands are read simultaneously.

|  |  | **Mean methyl levels** | **APC** | **Mean absolute difference** | **Strand bias** | **nCpGs** |
| --- | --- | --- | --- | --- | --- | --- |
| **Nanopolish** | **All CpGs** | 0.793 | 0.959 | 0.0471 | 0.0946 | 27,651,488 |
|  | **hq-CpGs** | 0.817 | 0.986 | 0.0314 | 0.0561 | 19,685,181 |
|  | **not hq-CpGs** | 0.735 | 0.882 | 0.0866 | 0.187 | 7,966,307 |
| **Guppy R9.4** | **All CpGS** | 0.763 | 0.973 | 0.0465 | 0.0644 | 27,659,182 |
|  | **Hq-CpGs** | 0.769 | 0.987 | 0.0383 | 0.0468 | 22,256,402 |
|  | **Not hq-CpGs** | 0.738 | 0.907 | 0.0814 | 0.138 | 5,402,780 |
| **Guppy R10.4** | **All CpGs** | 0.790 | 0.978 | 0.0339 | 0.0467 | 27,648,754 |
|  | **Hq-CpGs** | 0.792 | 0.991 | 0.0291 | 0.0339 | 22,893,522 |
|  | **Not hq-CpGs** | 0.781 | 0.901 | 0.0689 | 0.109 | 4,755,232 |
| **PacBio** | **All CpGs** | 0.782 | 0.970 | 0.0437 | NA^*^ | 27,527,663 |
|  | **Hq-CpGs** | 0.797 | 0.980 | 0.0380 | NA^*^ | 22,554,423 |
|  | **Not hq-CpGs** | 0.710 | 0.938 | 0.0705 | NA^*^ | 4,973,240 |

**^*^**Not reported for PacBio

**Table S5 Correlation analysis for CpGs excluded from the high-quality set of CpGs between oxBS methylation detection and Nanopolish, Guppy and PacBio.** For each group of CpGs we provide the number of CpG sites and pearson correlation coefficient, and additionally number of CpG-units for Nanopolish.

|  | **ONT Nanopolish** | | | **ONT Guppy R9.4** | | **ONT Guppy R10.4** | | **PacBio** | |
| --- | --- | --- | --- | --- | --- | --- | --- | --- | --- |
|  | Pearson r | Number of CpGs-units | Number of CpGs | Pearson r | Number of CpGs | Pearson r | Number of CpGs | Pearson r | Number of CpGs |
| **All CpGs** | 0.959 | 22,178,458 | 27,651,488 | 0.972 | 27,659,182 | 0.978 | 27,648,754 | 0.970 | 27,527,663 |
| **CpGs near sequence variants** | 0.922 | 2,949,801 | 4,026,250 | 0.940 | 3,713,303 | 0.934 | 3,711,812 | 0.929 | 3,696,786 |
| **CpGs within dark regions** | 0.698 | 522,262 | 698,627 | 0.716 | 695,535 | 0.736 | 688,246 | 0.691 | 611,068 |
| **CpGs with high/low coverage** | 0.722 | 673,474 | 896,984 | 0.702 | 581,583 | 0.752 | 796,000 | 0.918 | 1,409,536 |
| **CpGs with SB>0.2** | 0.828 | 2,800,184 | 3,044,175 | 0.854 | 1,577,810 | 0.626 | 850,227 | NA | NA |
| **CpGs with FRR<0.5** | 0.819 | 1,688,756 | 1,698,511 | NA | NA | NA | NA | NA | NA |
| **Hq CpGs** | 0.986 | 15,644,462 | 19,685,181 | 0.987 | 22,256,402 | 0.991 | 22,893,522 | 0.980 | 22,554,423 |
