# Supplementary material for: A comparison of methods for detecting DNA methylation from long-read sequencing of human genomes
Source: Genome Biol. 2024 Mar 11;25:69. doi: 10.1186/s13059-024-03207-9 (PMC10929077; doi:10.1186/s13059-024-03207-9)
Supplement: Supplementary file 3 — Additional file 3. Review history. [file 13059_2024_3207_MOESM3_ESM.docx]

**1^st^ round**

**Reviewer 1**

In this manuscript, the authors systematically evaluated the performance of CpG methylation detection from long-read sequencing in human genomes. Although there have been excellent works for benchmarking methylation-calling tools using nanopore sequencing, such as Yuen et al. 2021 (doi: 10.1038/s41467-021-23778-6) and Liu et al. 2021 (doi: 10.1186/s13059-021-02510-z), this study is distinct for the following reasons:
A. The authors compare the nanopore sequencing with oxBS using a large number of genomes/samples - 7,179 nanopore sequenced samples and 132 oxBS samples.
B. This study also includes evaluation of the latest ONT chemistry (R10.4) and sequencing technology (PacBio SMRT sequencing) with dozens of samples.
C. The authors have introduced some new quality filters (distance to sequence variants, abnormal sequencing coverage, strand bias, and fraction of reliable reads) that are rarely used in other studies, providing novel insights.
I do have some concerns and suggestions that I believe should be addressed or at least considered.

Major
1. The authors have sequenced a large number of samples/individuals/genomes, which is very valuable. However, the authors didn't clearly justify how these large number of samples distinguish this study from other works that only use several samples.
- Compared to benchmarking using several samples, are there any unique advantages by using large number of samples?
- Do the compared methods perform differently in samples of different ages or genders?

2. Will the raw sequencing data be made publicly available? How can readers reproduce this study? Additionally, following the instruction in Data availability section, I didn't find any summary statistics for this study in the website.

3. The authors did use (maybe) the most widely used tool - nanopolish, and the latest ONT official tool - Guppy6 with a Remora model, to call methylation from nanopore reads. However, there are other tools which are still under active development (such as deepsignal/deepsignal2 for R9.4, DeepMod/DeepMod2 for both R9.4 and R10.4) and were reported to have high performance.
- First, the authors should cite these works. Second, I suggest that the authors benchmark these methods, at least using part of their dataset.
- Regarding SMRT sequencing, I also suggest the authors benchmark the latest tool (ccsmeth), and cite related works (such as ccsmeth, modbamtools).
- The authors didn't clarify which tool/version they used for methylation detection from SMRT sequencing (primrose or jasmine?). There should be related information in the header of BAM files.

Minor
1. Page 6 Line 116 "the overall methylation levels per individual as the number of methylated CpGs divided with the total number of CpGs", how does a CpG count as a methylated CpG? Is here the same as Page 6 Line 137 (methylated 0.85-1)?

2. Page 6 Line 124, the numbers here (0.67 to 0.89, 0.076 to 0.22) seem don't match Fig. 1A and 1B.

3. Page 14 Line 290-291, I don't think in SMRT sequencing, the methylation detection is incorporated in the basecalling step.

4. Methylation phasing is now a hot topic with the advancement of long-read sequencing. I notice that the authors have discussed methylation phasing in Discussion section, also in Supplementary. But the notes "long range phasing" and figures (S13 and S14) in Supplementary were not mentioned/referenced in the main manuscript.

5. Following the above concern, it seems that in Page 28 Line 602, the "Per read performance evaluation" section is also not related to the Results section.
- Page 22 Line 689, it seems that ref. 22 is not complete, and there are no refs. 23-25.
- In the supplementary file, Page 10 "in bimodal distribution (Fig. 4., Additional File 1: Fig. S12).", there is no Fig. 4 in the main manuscript, and Fig. S12 is also not mentioned/referenced in the main manuscript.

6. The authors have oxBS data for some samples. Since ONT now has Remora R10.4 models for 5hmC detection, could the authors also benchmark how well the remora model performs in detecting 5hmC using some of their samples?

**Reviewer 2**

This well-written manuscript compares two methods of detecting CpG methylation via long read sequencing -- nanopore sequencing and single molecule real-time (SMRT) sequencing - to a baseline approach, oxidative bisulfite sequencing (oxBS), with the main focus on evaluating computational methods for analyzing nanopore sequencing data. Earlier studies have evaluated several different methods for analyzing nanopore sequencing data on a very small number of samples; the current manuscript focuses on only two of these methods but includes data from a large number of samples. The analyses presented are carefully described and appear valid, and the conclusions concisely present 5 key takeaways that will have practical value for readers planning nanopore sequencing studies. Some concerns and suggestions are listed below:

1) Please discuss more explicitly what the work in the submitted manuscript adds to the field beyond the evaluations cited in refs 20-21, which each present comparisons of 7 analytic tools, including a consensus approach (METEORE). Could highlight innovations such as use of latest chemistry, consideration of strand bias, etc. Also, reference 21 (cited as a biorxiv paper) was published in Nature Communications in 2021 - this outdated citation should be corrected.

2) Although the oxBS data provide a baseline for comparison to a well-known technology, there is no comparison to a gold standard where the true values are known, so this work provides more of a comparison than an evaluation. That said, the comparisons are performed thoughtfully and the authors do make a convincing case that Guppy outperforms Nanopolish based on strand bias.

3) Because the SMRT sequencing was performed on a different set of samples, the comparisons between this technology and others in Table 1 are comparisons between different sets of samples that are not matched on variables like age, gender or smoking status.

4) More descriptive data comparing the three sequencing technologies would be of interest to many readers; although median coverage is presented for each, read length is not discussed. Plots showing the distributions of coverage and read length could be provided to compare the technologies and give the readers a better sense of the data

5) Why was Guppy applied to a smaller fraction of the samples? How were these samples chosen?

6) Line 417: "even for samples with higher error rate": this is a confusing sentence to lead the Discussion with because this information is barely discussed in the manuscript, other than a brief reference to the supplement (which most casual readers won't access). If this is worth mentioning in the discussion, is it worth providing a more detailed summary of the analysis in the results section?

Minor concerns:

1) Refs 12 and 22 are the same reference, and neither includes information on where this work can be accessed. Is this a work in progress (unpublished manuscript)? If so, should be noted as such.

2) In Fig 1E the stacked portions of the bars do not appear to be in any logical order (they do not represent an ordering based on abs diff). This should be easy to correct.

3) Line 218: should explicitly state here what cutoffs were used to define high strand bias and low FRR for elimination of low-quality CpGs. These are suggested above and in Fig S5 but not explicitly stated, and this way the reader doesn't have to hunt for them.

4) Some of the nonstandard abbreviations are a bit unwieldy and can lead to confusion, particularly 5-mCpG. Why not use the standard abbreviation 5mC? PCAPC is also confusing - the "per CpG" part is probably not necessary to state, as it is quite common to estimate an average methylation proportion across all CpGs.

**Authors’ response**

Reviewer #1: In this manuscript, the authors systematically evaluated the performance of CpG methylation detection from long-read sequencing in human genomes. Although there have been excellent works for benchmarking methylation-calling tools using nanopore sequencing, such as Yuen et al. 2021 (doi: 10.1038/s41467-021-23778-6) and Liu et al. 2021 (doi: 10.1186/s13059-021-02510-z), this study is distinct for the following reasons:

A. The authors compare the nanopore sequencing with oxBS using a large number of genomes/samples - 7,179 nanopore sequenced samples and 132 oxBS samples.

B. This study also includes evaluation of the latest ONT chemistry (R10.4) and sequencing technology (PacBio SMRT sequencing) with dozens of samples.

C. The authors have introduced some new quality filters (distance to sequence variants, abnormal sequencing coverage, strand bias, and fraction of reliable reads) that are rarely used in other studies, providing novel insights.

I do have some concerns and suggestions that I believe should be addressed or at least considered.

Major

1. The authors have sequenced a large number of samples/individuals/genomes, which is very valuable. However, the authors didn't clearly justify how these large number of samples distinguish this study from other works that only use several samples.

- Compared to benchmarking using several samples, are there any unique advantages by using large number of samples?

- Do the compared methods perform differently in samples of different ages or genders?

Reply:

We have added short paragraph in the „Background“ section where we explain the benefits of the large number of samples sequenced in our study:

„By analysing large numbers of genomes, we accurately assess the reliability of CpG methylation predictions from nanopore long-read sequencing developed by ONT and introduce generalized quality filters that can be applied to other cohorts, providing guidance for researchers performing 5-mCpG studies based on long-read sequencing.“

Additionally, we have rewritten the „Discussion“ section, where we discuss in more detail what has previously been done in the field. We now specifically discuss how large number of genomes are beneficial on page 18, line 406:

„By using a large cohort, we can reduce the risk of drawing erroneous conclusions due to random variability and have more robustness to outliers. Our study extends beyond previous studies by showing that the quantification of methylation varies in quality between CpG. We filter out unreliable CpGs and define a set of hq-CpGs that led to significantly improved accuracy while still providing comprehensive analysis of over 70% of autosomal CpG sites. By tuning the quality attributes on a large cohort, they are more likely to be representative of the broader population and therefore generalized...“.

We did not consider whether the methods performed differently with respect to age and sex, but we have noted that this may influence our results, see page 14, lines 322-324.

We have also made a note in Supplementary chapter 1.5, page 12.

See in more detail in comment #3 from reviewer 2.

2. Will the raw sequencing data be made publicly available? How can readers reproduce this study? Additionally, following the instruction in Data availability section, I didn't find any summary statistics for this study in the website.

Reply: The raw sequencing data will not be made publicly available. We have added the summary statistics files to the following website, such that all calculations can be validated and compared to other datasets: https://download.decode.is/folder/efb18487- c8f8-40fc-ad83-41170a98cf18

This will be made available under www.decode.com/summarydata/ if accepted.

See more info under „Supplementary Notes – Chapter 2: Supplementary data description“.

3. The authors did use (maybe) the most widely used tool - nanopolish, and the latest ONT official tool - Guppy6 with a Remora model, to call methylation from nanopore reads. However, there are other tools which are still under active development (such as deepsignal/deepsignal2 for R9.4, DeepMod/DeepMod2 for both R9.4 and R10.4) and were reported to have high performance.

- First, the authors should cite these works. Second, I suggest that the authors benchmark these methods, at least using part of their dataset.

- Regarding SMRT sequencing, I also suggest the authors benchmark the latest tool (ccsmeth), and cite related works (such as ccsmeth, modbamtools).

- The authors didn't clarify which tool/version they used for methylation detection from SMRT sequencing (primrose or jasmine?). There should be related information in the header of BAM files.

Reply: We have added the suggested references.

When we started nanopore sequencing in 2017, Nanopolish was the most promising tool for methylation detection. Yuen et al. (1) show the benefits of combining predictions from two or more tools, so we decided to add another methylation detection tool to our pipeline. Based on the paper, and another paper by Liu et al (2), we decided that guppy would be the best fit for our needs, mainly due to two reasons:

Coputational resources vs. performance: Nanopolish and Guppy required both the least amount of CPU time and exhibited the lowest peak memory usage for highly accurate methylation detection.

Accessibility: Many of the other tools required file formats that were no longer used in our pipeline and based on the pipeline design, Guppy was the most convenient tool as it was already set up in our pipeline for basecalling.

DeepSignal is both more accurate and can detect up to 4-6% more CpGs than Nanopolish and Guppy (3), however running it on large scale is not ideal due to CPU time and memory usage. Further, the tool requires write access to the fast5 files and splitting multi fast5, meaning a lot of data being copied and a few time consuming steps applied before the actual methylation detection. These steps make the program impractical to run at scale in its current form.

We tested 10 samples with DeepSignal2 and came to the same conclusion, that it is highly accurate but not ideal for running on the scale that we are working on.

We have updated the description of methylation detection from SMRT-sequencing on page 13, lines 301-303 as follows:

„We use primrose for methylation detection, which is applied on the sequencer after basecalling.“

Minor

1. Page 6 Line 116 "the overall methylation levels per individual as the number of methylated CpGs divided with the total number of CpGs", how does a CpG count as a methylated CpG? Is here the same as Page 6 Line 137 (methylated 0.85-1)?

Reply: Here we refer to the binary status of each CpG within sequences to be classified as either methylated or unmethylated based on a criteria unique for each tool.

We have updated the manuscript for clarification on page 6, lines 117-120:

„The overall methylation levels per individual was computed by counting the number of times a methylated status was assigned to a CpG detected in sequences obtained from a given DNA sample to then divide this number by the total number of times we were able to assign a methylation status (unmethylated or methylated) to CpG sites in sequences obtained from that same DNA sample.“

2. Page 6 Line 124, the numbers here (0.67 to 0.89, 0.076 to 0.22) seem don't match Fig. 1A and 1B.

Reply: We have updated as follows (now page 6, line 131):

„We found that the correlation varied from 0.71 to 0.94 and the MAD from 0.076 to 0.14.“

3. Page 14 Line 290-291, I don't think in SMRT sequencing, the methylation detection is incorporated in the basecalling step.

Reply: We have rephrased and added the following sentence to that section (now page 13, lines 301-303):

„We use primrose for methylation detection, which is performed by the sequencer after basecalling.“

4. Methylation phasing is now a hot topic with the advancement of long-read sequencing. I notice that the authors have discussed methylation phasing in Discussion section, also in Supplementary. But the notes "long range phasing" and figures (S13 and S14) in Supplementary were not mentioned/referenced in the main manuscript.

Reply: We have updated the manuscript and removed the phasing from the Supplementary Notes. We cite our unpulished manuscript in both results and discussion (4), where we use phased methylation to investigate the relationship between sequence variants, gene expression and CpG methylation.

5. Following the above concern, it seems that in Page 28 Line 602, the "Per read performance evaluation" section is also not related to the Results section.

- Page 22 Line 689, it seems that ref. 22 is not complete, and there are no refs. 23-25.

- In the supplementary file, Page 10 "in bimodal distribution (Fig. 4., Additional File 1:

Fig. S12).", there is no Fig. 4 in the main manuscript, and Fig. S12 is also not mentioned/referenced in the main manuscript.

Reply: First, we agree and removed the „Per read performance evaluation“ chapter.

Second, we‘ve added the missing references and removed 22 as it was duplicated (See Reviewers 2 minor comment #2).

Third, I‘ve updated to Figure 3, and added a reference to Fig. S12.

6. The authors have oxBS data for some samples. Since ONT now has Remora R10.4 models for 5hmC detection, could the authors also benchmark how well the remora model performs in detecting 5hmC using some of their samples?

Reply: We agree that this is a very interesting project and would add to the novelty of this study. In the future we expect to add 5hmC to our analysis pipeline. We argue however that it is not within the scope of this manuscript for two main reasons:

To be able to compare the 5hmC we would have to BS sequence the samples as well and compute the differences in measured 5-mCpG rates between oxBS and BS; the residual rate is the 5hmC rate. We do not currently have any BS data inhouse but will most likely add in the future.

The level of 5hmC in blood derived DNA is extremely low (0.004%) (5), which makes it less relevant for our current study which is exclusively based on DNA samples from whole blood.

Reviewer 2 reports and replies

Reviewer #2: This well-written manuscript compares two methods of detecting CpG methylation via long read sequencing -- nanopore sequencing and single molecule real-time (SMRT) sequencing - to a baseline approach, oxidative bisulfite sequencing (oxBS), with the main focus on evaluating computational methods for analyzing nanopore sequencing data. Earlier studies have evaluated several different methods for analyzing nanopore sequencing data on a very small number of samples; the current manuscript focuses on only two of these methods but includes data from a large number of samples. The analyses presented are carefully described and appear valid, and the conclusions concisely present 5 key takeaways that will have practical value for readers planning nanopore sequencing studies. Some concerns and suggestions are listed below:

1) Please discuss more explicitly what the work in the submitted manuscript adds to the field beyond the evaluations cited in refs 20-21, which each present comparisons of 7 analytic tools, including a consensus approach (METEORE). Could highlight innovations such as use of latest chemistry, consideration of strand bias, etc. Also, reference 21 (cited as a biorxiv paper) was published in Nature Communications in 2021 - this outdated citation should be corrected.

Reply: Thank you, we have now more explicitly stated how our study improves upon previous studies in the discussion section, see page 18, where we write:

„By using a large cohort, we can reduce the risk of drawing erroneous conclusions due to random variability and have more robustness to outliers. Our study extends beyond previous studies by showing that the quantification of methylation varies in quality between CpG. We filter out unreliable CpGs and define a set of hq-CpGs that led to significantly improved accuracy while still providing comprehensive analysis of over 70% of autosomal CpG sites. By tuning the quality attributes on a large cohort, they are more likely to be representative of the broader population and therefore generalized. The most significant improvement in APC was achieved by removing dark regions and regions with abnormal read coverage. APC between all pairs methods was lower for dark regions, suggesting that these regions are less reliable for all methods. Filtering out CpGs located ≤5 bp from sequence variants is necessary for Nanopolish because of the way the algorithm is designed, but this is not a necessary filtering criteria for other methods. All methods however, likely benefit from filter on CpGs where a sequence variant occurs on either the cytosine or guanine base within the CpG motif itself. We note, that the aforementioned filters may need to be reevaluated depending on each project‘s needs.“

We then move on to discuss the use of the latest R10.4 nanopore flowcell as suggested by the reviewer. On page 18, we write:

„We further show that Guppy applied to R10.4 flowcells with updated chemistry, resolves some of the problems seen in the earlier versions of Guppy applied to R9.4 flowcells, such as strand bias and results in larger set of hq-CpGs. We report that for SMRT-sequencing the methylation predictions (12,13) never reach either fully unmethylated, or fully methylated state. Changes to the model, such that the predictions do not regress away from either of these two extremes may be beneficial. “

We were aware of the published paper in Nature Communications in 2021 but failed to update it from a biorxiv paper in our database. We have now added the correct reference.

In response to the reviewers comment, we decided to rewrite the Discussion section to talk more about previous work in the field, the novelty of our paper and what we add to the field.

2) Although the oxBS data provide a baseline for comparison to a well-known technology, there is no comparison to a gold standard where the true values are known, so this work provides more of a comparison than an evaluation. That said, the comparisons are performed thoughtfully and the authors do make a convincing case that Guppy outperforms Nanopolish based on strand bias.

Reply: We have updated the language in several places to make the readers aware of this.

For an example, page 4, line 85-88:

„In this study, we present a systematic comparison of 5-mCpG methylation detection tools for nanopore sequencing (ONT) of 7,179 DNA samples, including 22 samples sequenced with the latest nanopore flowcell chemistry, 132 oxBS sequenced samples from the same blood draw, and 50 samples sequenced using SMRT technology (PacBio).“

On page 14, line 317:

„We averaged the 5-mCpG rates over all samples and compared the APC correlation coefficient between all five methods (SMRT, R9.4-Guppy, R10.4- Guppy, R9.4-Nanopolish and oxBS) and the absolute difference between 5-mCpG rates and oxBS (Table 1A).“

3) Because the SMRT sequencing was performed on a different set of samples, the comparisons between this technology and others in Table 1 are comparisons between different sets of samples that are not matched on variables like age, gender or smoking status.

Reply: We agree and are aware that this is a drawback of our study, which is why we state the following: „The difference may be partially explained by differences in age, gender or smoking status of the samples (Additional file 1: Tab. S3).“

We further show that the other subsets are not all equally distributed between gender, age groups and smokers. Moreover, the effect of these attributes involve certain and relatively few CpGs, considering the >15 million measured CpGs, so we assume that these attributes will not have a large effect when comparing all CpGs. Additionally, the effect size of age on the 5-mCpG rate, if at all present, tends to be rather subtle. The effect size of gender, however, is mainly found on chromosome X where it is large, and it is this sex-specific methylation of chromosome X that is one reason why we choose to restrict to autosomes in our study. Smoking, on the other hand, can have a large effect size on 5-mCpG rates, but the effects tends to be restricted to relatively few and specific CpGs. For these reasons, we do not expect that these attributes will interfere much with our overall comparison of millions of CpGs based on measuring the APC. We have added a note on this issue in the Supplementary, Chapter 1.5, page 12:

„We note, however, that some of the differences in APC and MAD observed between methods may be due to differences in age, gender or smoking status of the samples. The effect of these attributes involve relatively few and specific CpGs, considering the >15 million measured CpGs, and the effects tend to be subtle. For this reason, we assume that these attributes will not have a large effect when comparing all CpGs based on the APC parameter.“

4) More descriptive data comparing the three sequencing technologies would be of interest to many readers; although median coverage is presented for each, read length is not discussed. Plots showing the distributions of coverage and read length could be provided to compare the technologies and give the readers a better sense of the data

Reply: We agree that this is a good addition to our paper and have added Supplementary Figure S9:

Figure S9 Comparison of sequencing statistics for long-read sequencing techniques. A Boxplot showing the sequencing coverage (y-axis) for each method (x-axis). B Box plot showing the N50 (y-axis) distribution for each method (x-axis). C Box plot showing the percentage error rate (y-axis) for each method (x-axis). The centre line (solid black) shown in each box represent the median; the box limits represent upper and lower quartile, whiskers represent 1.5x interquartile range.

We also added a summary to page 13, lines 295-301:

„We SMRT-sequenced whole- blood samples from 50 individuals on 170 flowcells to average sequencing coverage of 28.5x per sample (range 13.6-41.7x), which was higher than for nanopore R9.4 and R10.4 sequencing methods (Additional file 1: Fig. S9A). The average N50, defined as the length of the sequence read at 50th percentile of the total sequence read length, was similar for SMRT and nanopore R9.4 and R10.4 sequencing methods (Additional file 1: Fig. S9B), but the average sequencing error rate was lower for SMRT- sequencing than either of the two nanopore sequencing methods, or 1.12% (range 1.02-1.31%, Additional file 1: Fig. S9C).“

5) Why was Guppy applied to a smaller fraction of the samples? How were these samples chosen?

Reply: Guppy has been used for the basecalling of all samples but the methylation detection step was added to the pipeline mid 2022. All new samples or samples that were re-basecalled after that time point were additionally methylation called using Guppy.

As Guppy has been under constant developement in the recent years, it is simply impossible for us to be up to date with the newest basecaller applied to the whole dataset (over 7000 samples). We argue that it is not important to call Guppy again, including the methylation step, on all samples at this point, but as soon as there will be significant update we will run the methylation step as well.

6) Line 417: "even for samples with higher error rate": this is a confusing sentence to lead the Discussion with because this information is barely discussed in the manuscript, other than a brief reference to the supplement (which most casual readers won't access). If this is worth mentioning in the discussion, is it worth providing a more detailed summary of the analysis in the results section?

Reply: We agree and have removed this sentence. We have rewritten the Discussion section, see more details in comment #1 from this same reviewer.

Minor concerns:

1) Refs 12 and 22 are the same reference, and neither includes information on where this work can be accessed. Is this a work in progress (unpublished manuscript)? If so, should be noted as such.

Reply: We‘ve removed duplicated refereneces and updated to unpublished manuscript. I‘ve further added missing references.

2) In Fig 1E the stacked portions of the bars do not appear to be in any logical order (they do not represent an ordering based on abs diff). This should be easy to correct.

Reply: We agree and have now rearranged the bars such that they are in logical order. We further updated the order of the plots (moved F to A) to match the text.

3) Line 218: should explicitly state here what cutoffs were used to define high strand bias and low FRR for elimination of low-quality CpGs. These are suggested above and in Fig S5 but not explicitly stated, and this way the reader doesn't have to hunt for them.

Reply: We‘ve updated the text as follows:

„Consequently, we define problematic CpG-units as being within of dark regions, within 5 bp distance from a SNP, having coverage ≤0.5 times or ≥1.5 times the average coverage, strand bias≥0.2 and FRR≤0.5.“

4) Some of the nonstandard abbreviations are a bit unwieldy and can lead to confusion, particularly 5-mCpG. Why not use the standard abbreviation 5mC?

PCAPC is also confusing - the "per CpG" part is probably not necessary to state, as it is quite common to estimate an average methylation proportion across all CpGs.

Reply: We agree and have updated the PCAPC abbrevation to APC in the text. For an example on page 5, line 113:

„We refer to this correlation as per CpG average Pearson correlation (APC).“

On page 8, line 179-181:

„We found that CpG units located within 5 bp of a sequence variant had a lower APC (r=0.9219; 95%CI:0.9218-0.9221) than other CpG units (r=0.96560, 95%CI:0.96557-0.96563) (Fig. 2A).“

On page 11, lines 250-252:

„The methylation calls from Guppy and Nanopolish were highly correlated, with a APC of 0.96558 (95%CI:0.96555-0.96561) for the full set of CpGs. Guppy had higher APC with oxBS data (r=0.97256; 95%CI: 0.97255-0.97259) than Nanopolish (r=0.9594; 95%CI:0.9594-0.9595).“

We selected 5-mCpG due to it being more accurate than 5mC which does not distinguish between 5mCpG and 5-mCpH where H is any other base than G.

References

1.Yuen ZWS, Srivastava A, Daniel R, McNevin D, Jack C, Eyras E. Systematic benchmarking of tools for CpG methylation detection from nanopore sequencing. Nat Commun. 2021;12(1).

2.Liu Y, Rosikiewicz W, Pan Z, Jillette N, Wang P, Taghbalout A, et al. DNA methylation-calling tools for Oxford Nanopore sequencing: a survey and human epigenome-wide evaluation. Genome Biol. 2021;22(1).

3.Liu Y, Rosikiewicz W, Pan Z, Jillette N, Wang P, Taghbalout A, et al. DNA methylation-calling tools for Oxford Nanopore sequencing: a survey and human epigenome-wide evaluation. Genome Biol. 2021;22(1).

4.Stefansson OA, Sigurpalsdottir BD, Rognvaldsson S, Halldorsson GH, Juliusson K, Sveinbjornsson G, et al. The correlation between CpG methylation and gene expression is driven by sequence variants. [Unpublished manuscript].

5.Gilat N, Tabachnik T, Shwartz A, Shahal T, Torchinsky D, Michaeli Y, et al. Single- molecule quantification of 5-hydroxymethylcytosine for diagnosis of blood and colon cancers. Clin Epigenetics. 2017;9(1).

**2^nd^ round**

**Reviewer 1**

I thank the authors for their hard work. Most of my concerns are addressed. I only have one concern now.
- I understand that this cohort cannot be made publicly available due to ethical or other considerations. However, could these data be made available under controlled access? I would appreciate further explanation if the authors cannot make their data public. Also, I still cannot find any summary statistics in the website provided.

**Reviewer 2**

The authors have addressed my comments from the previous submission. I am satisfied with the revision, though I am confused about the coverage recommendations made on lines 132-137 and how they correspond to the information in the figures. My interpretation of Figs 1B-C would be that 12x is insufficient and 20x would be a more appropriate recommendation, given levels of both APC and MAD at 12x vs. >20x coverage. And my interpretation of Fig 1D would be that 25x is more conservative than necessary, since performance appears as good or better for the bin with coverages of 21-24x. Perhaps I am misunderstanding something about these figures, but clarification on this point would be helpful.

**Authors’ response**

Reviewer #1: I thank the authors for their hard work. Most of my concerns are addressed. I only have one concern now.

- I understand that this cohort cannot be made publicly available due to ethical or other considerations. However, could these data be made available under controlled access? I would appreciate further explanation if the authors cannot make their data public. Also, I still cannot find any summary statistics in the website provided.

We apologize for the broken summary statistics link. The protocol is to send a private link to reviewers and not making the data public until the paper is accepted. Unfortunately, by mistake the link was set to expire within 30 days. Now the data should be available from our website and accessible to anyone:

www.decode.com/summarydata/ and on the following zenodo page:

10.5281/zenodo.10683994.

To answer your concerns, we have upated the „Availability of data and materials“

section on page 28:

„Access to the raw Icelandic sequence data is available on request from Kári Stefánsson at the premises of deCODE genetics. The data are not publicly available because of Icelandic state law. All analyses in the manuscript can be recreated using the 5-mCpG rates summary statistics, which can be downloaded from our website: www.decode.com/summarydata/ (39).“

Reference: 39. Sigurpálsdóttir BD, Stefansson OA, Holley G, Beyter D, Zink F, Hardarson MÞ, Sverrisson SÞ, Kristinsdottir NK, Magnusdottir DN, Magnusson OÞ, Gudbjartsson DF, Halldorsson BV, Stefansson K. A comparison of methods for detecting DNA methylation from long-read sequencing of human genomes. Datasets. Zenodo. https://doi.org/10.5281/zenodo.10683994 (2024).

Reviewer #2: The authors have addressed my comments from the previous submission. I am satisfied with the revision, though I am confused about the coverage recommendations made on lines 132-137 and how they correspond to the information in the figures. My interpretation of Figs 1B-C would be that 12x is insufficient and 20x would be a more appropriate recommendation, given levels of both APC and MAD at 12x vs. >20x coverage. And my interpretation of Fig 1D would be that 25x is more conservative than necessary, since performance appears as good or better for the bin with coverages of 21-24x. Perhaps I am misunderstanding something about these figures, but clarification on this point would be helpful.

We agree with the reviewer that there are some inconsistencies between the figures and the text. We also see how the text can be misunderstood, as we mainly wanted to make the readers aware of the fact that sequencing coverage of 12x is already quite good but as stated by reviewer 20x or higher is highly recommended for the best results.

We further agree that 25x per CpG is too conservative. To clarify we have updated the text on page 6:

„The correlation was notably higher and MAD lower for high coverage samples, indicating that sequencing coverage of approximately 12x or more per sample is advisable for accurate methylation detection and sequencing at 20x or greater yields even more accurate results (Fig. 1B, 1C). We then calculated the Pearson correlation for each sample, for all CpG sites with high sequence coverage (greater than 25x) supporting a minimum nanopore sequencing depth of a CpG unit as 20x for obtaining a highly reliable measurement of its 5-mCpG rate (Fig. 1D).„
